# Early antiviral CD4^+^ and CD8^+^ T cells are associated with upper airway clearance of SARS-CoV-2

**DOI:** 10.1172/jci.insight.186078

**Published:** 2024-12-20

**Authors:** Sydney I. Ramirez, Paul G. Lopez, Farhoud Faraji, Urvi M. Parikh, Amy Heaps, Justin Ritz, Carlee Moser, Joseph J. Eron, David Wohl, Judith Currier, Eric S. Daar, Alex Greninger, Paul Klekotka, Alba Grifoni, Daniela Weiskopf, Alessandro Sette, Bjoern Peters, Michael D. Hughes, Kara W. Chew, Davey M. Smith, Shane Crotty, for the Accelerating COVID-19 Therapeutic Interventions and Vaccines-2 (ACTIV-2)/A5401 Study Team

**Affiliations:** 1 Center for Vaccine Innovation, La Jolla Institute for Immunology, La Jolla, California, USA.; 2 Division of Infectious Diseases and Global Public Health, Department of Medicine, and; 3 Department of Otolaryngology-Head and Neck Surgery, UCSD, La Jolla, California, USA.; 4 Division of Infectious Diseases, Department of Medicine, University of Pittsburgh School of Medicine, Pittsburgh, Pennsylvania, USA.; 5 Center for Biostatistics in AIDS Research, Harvard T.H. Chan School of Public Health, Boston, Massachusetts, USA.; 6 Department of Medicine, University of North Carolina at Chapel Hill School of Medicine, Chapel Hill, North Carolina, USA.; 7 Division of Infectious Diseases, Department of Medicine, David Geffen School of Medicine at UCLA, Los Angeles, California, USA.; 8 Lundquist Institute at Harbor-UCLA Medical Center, Torrance, California, USA.; 9 Department of Laboratory Medicine and Pathology, University of Washington Medical Center, Seattle, Washington, USA.; 10 Eli Lilly and Company, San Diego, California, USA.; 11 The ACTIV-2/A5401 Study Team is detailed in Supplemental Acknowledgments.

**Keywords:** Immunology, Infectious disease, Adaptive immunity, Cellular immune response, T cells

## Abstract

T cells are involved in protective immunity against numerous viral infections. Data regarding functional roles of human T cells in SARS-CoV-2 (SARS2) viral clearance in primary COVID-19 are limited. To address this knowledge gap, we assessed samples for associations between SARS2 upper respiratory tract viral RNA levels and early virus-specific adaptive immune responses for 95 unvaccinated clinical trial participants with acute primary COVID-19 aged 18–86 years old, approximately half of whom were considered at high risk for progression to severe COVID-19. Functionality and magnitude of acute SARS2-specific CD4^+^ and CD8^+^ T cell responses were evaluated, in addition to antibody responses. Most individuals with acute COVID-19 developed SARS2-specific T cell responses within 6 days of COVID-19 symptom onset. Early CD4^+^ T cell and CD8^+^ T cell responses were polyfunctional, and both strongly associated with reduced upper respiratory tract SARS2 viral RNA, independent of neutralizing antibody titers. Overall, these findings provide evidence for protective roles for circulating SARS2-specific CD4^+^ and CD8^+^ T cells during acute COVID-19.

## Abstract 2

A knowledge gap about the role of T cells in protection against SARS-CoV-2 remains, with limited human studies of antiviral T cell responses to COVID-19.

## Introduction

Since the emergence of SARS-CoV-2 (SARS2) as a novel human pathogen, much has been learned about protective immunity to SARS2 in the contexts of infection and COVID-19 vaccines. Serologic correlates of protection have been established for vaccines (1–8). In the context of prophylaxis, virus neutralization by monoclonal antibodies (mAbs) has been demonstrated as one mechanism of protective immunity (9–12). In contrast, in the context of infection, the relative importance of immune system compartments may differ, due to the substantial differences in kinetics of primary versus memory immune responses and lack of preexisting antibodies. Studies of primary adaptive immunity to acute SARS2 infection offer a key opportunity to evaluate early humoral and cellular immune responses and their individual contributions to protection.

The global population rapidly developed widespread cellular and humoral immunity to SARS2 as a result of both vaccination and infection. However, key gaps in our understanding of human primary immune responses to SARS2 remain. Cellular immunity may be required for viral control and clearance during acute infection (13–17). Although acute and memory T cell responses to SARS2 infection (18–24) and COVID-19 vaccines clearly occur (25–32), evidence of functional protective roles of T cells has been limited in humans (16, 33), particularly from assessing nonhospitalized COVID-19 cases (34–36). Due to the combined difficulties of identifying early acute COVID-19 cases and recruiting those individuals and technical challenges of measuring virus-specific T cell responses and viral loads concomitantly, most acute T cell studies have been limited to small cohorts, including hospitalized cases, sometimes relatively late in disease, and often without viral load measurements (21, 24, 37–39). In a controlled SARS-CoV-2 human challenge study of 18 young adults with a mean age of 22 years old, early circulating SARS2-specific CD8^+^ T cells were associated with SARS2 viral clearance (36). There is evidence for cellular immunity contributing to SARS2 protection in nonhuman primate models (16, 17). Breakthrough infections represent a different context for studying protective immunity in humans, in which there is evidence for contributions of memory T cells (40, 41). Given expanded interest in next-generation COVID-19 and pan-sarbecovirus vaccines with T cell–specific components (42, 43), or vaccines that are entirely T cell based (30, 44, 45), future vaccine designs and clinical trial designs would benefit from better fundamental understanding of T cell protective immunity to COVID-19 in humans (30, 34).

## Results

Longitudinal data were collected for 95 individuals with primary SARS2 infection in 2020, before the availability of COVID-19 vaccines, with sampling including nasopharyngeal (NP) swabs for SARS2 RNA, serum, and peripheral blood mononuclear cells (PBMCs), all collected in the context of a randomized, controlled clinical trial (ACTIV-2/A5401, ClinicalTrials.gov NCT04518410). Herein, serum antibody, SARS2-specific CD4^+^ T cell, and SARS2-specific CD8^+^ T cell response measurements are reported, representing the largest and most comprehensive acute viral and antigen-specific immune response data set of its kind.

**Figure 1:**
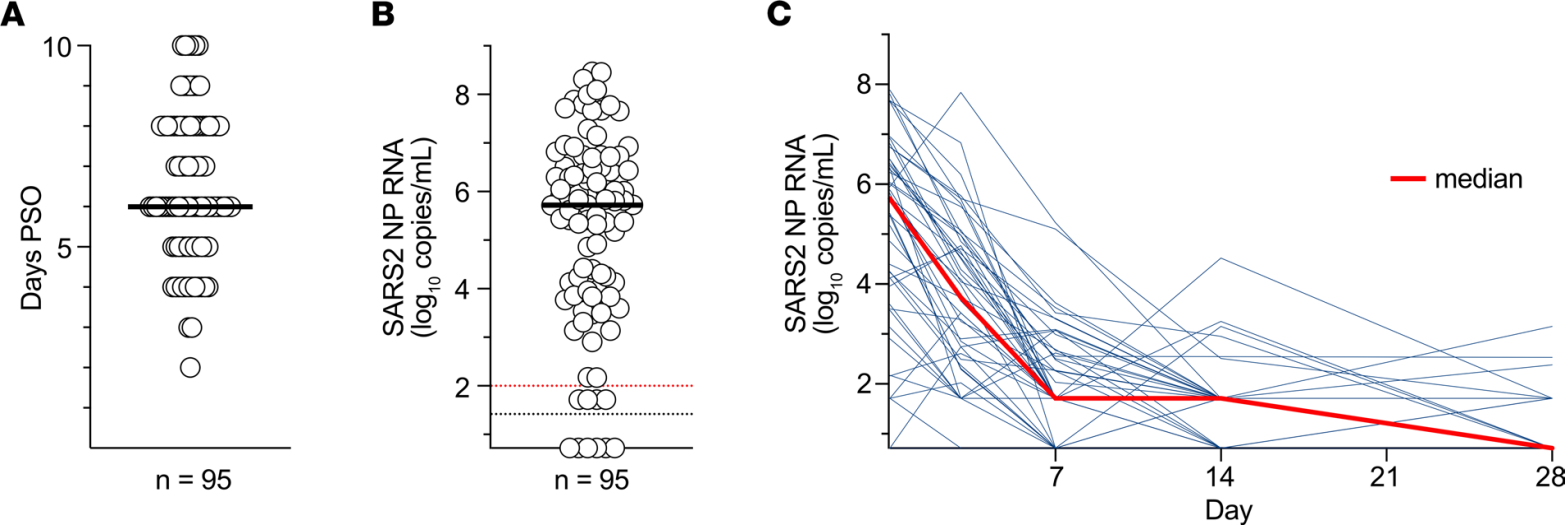
SARS2 NP RNA levels during acute COVID-19 and longitudinally. (**A**) Median days post-symptom onset (PSO) from start of COVID-19 symptoms to study entry (study day 0) for all participants. (**B**) SARS-CoV-2 NP RNA by quantitative reverse transcription polymerase chain reaction for all participants prior to treatment on study day 0. Dotted black line shows limit of detection (LOD). Dotted red line shows limit of quantification (LOQ). Values > LOQ were considered positive. Bar = median. (**C**) Longitudinal SARS-CoV-2 NP RNA data for the placebo group (*n* = 49) for study days 0, 7, 14, and 28. Median for each time point in red.

All participants were enrolled within 7 days from positive SARS2 testing and 10 days from COVID-19 symptom onset (20, 46). Individuals studied herein were among those randomized to receive either 700 mg of the mAb bamlanivimab or placebo (saline) intravenously (Table 1) on study day 0 (median of 6 days post-symptom onset, PSO; Figure 1A). The 46 bamlanivimab treatment and 49 placebo individuals were similar with respect to baseline characteristics, such as age, biological sex at birth, risk for progression to severe COVID-19, and time PSO at randomization (Table 1 and Supplemental Figure 1A; supplemental material available online with this article; https://doi.org/10.1172/jci.insight.186078DS1) (20, 46). Peripheral blood was collected prior to mAb or placebo administration on study day 0. NP swabs were collected from all 95 participants by trained study staff before treatment on study day 0, with SARS2 viral RNA detectable in the NP swabs of nearly all individuals (89%) (Figure 1B) and no significant difference in SARS2 NP RNA between the bamlanivimab (treatment) and placebo groups (Supplemental Figure 1B). SARS2 NP RNA at study day 0 was not associated with participant age (*r* = 0.16, *P* = 0.12, Supplemental Figure 1C). SARS2 NP RNA levels declined over time (Figure 1C), as expected (47–49), and as previously reported for the full ACTIV-2/A5401 cohort (46).

**Figure 2:**
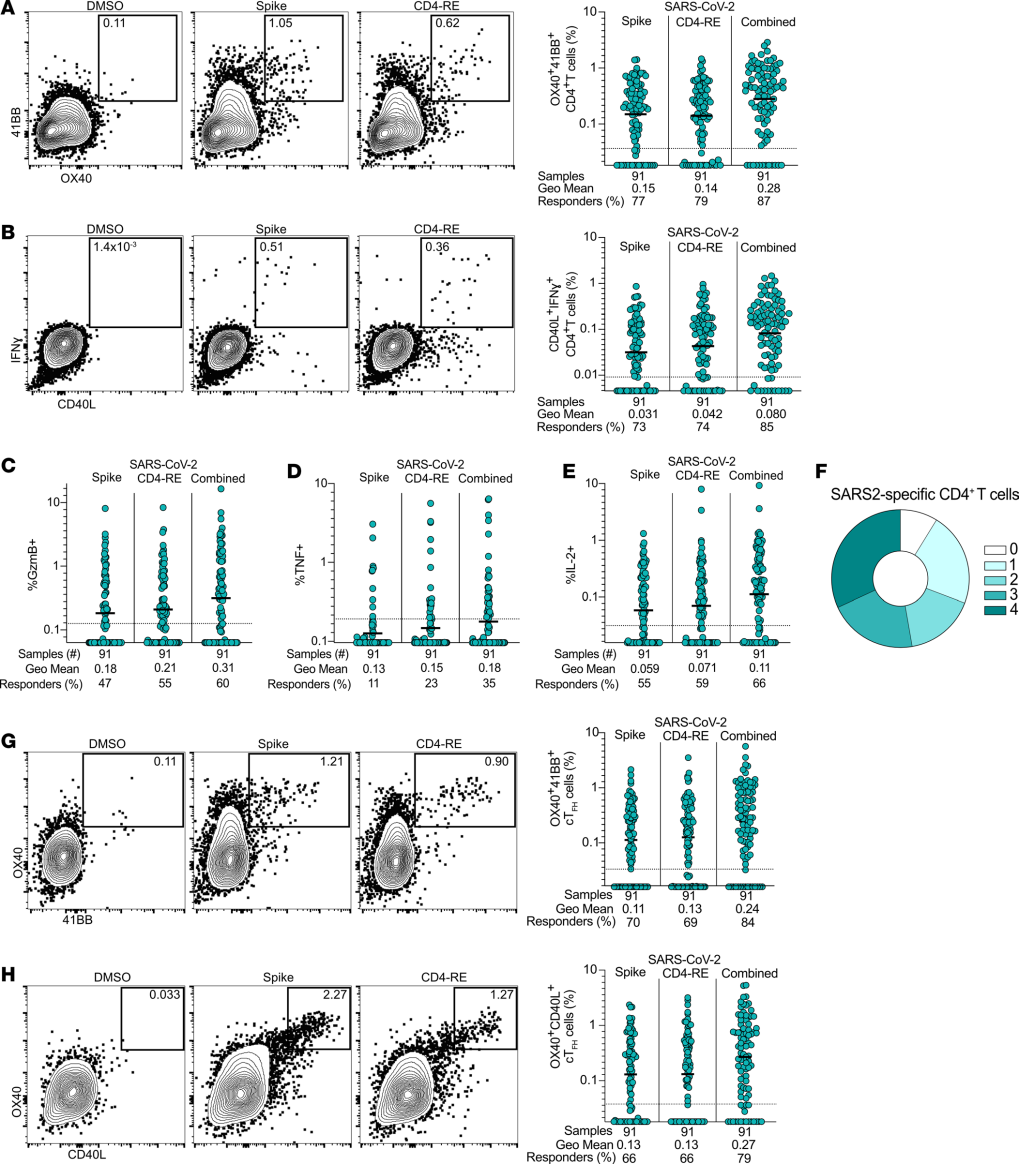
Antigen-specific CD4^+^ T cell responses to primary SARS2 infection and acute COVID-19. (**A** and **B**) Representative flow cytometry plots and frequency of SARS2-specific CD4^+^ T cells to DMSO (negative control), S, and CD4-RE MP stimulation conditions (Combined = sum of S + CD4-RE responses; see Methods for additional details) by (**A**) AIM using surface OX40 and 41BB coexpression and (**B**) IFN-γ ICS among surface CD40L^+^ cells. (**C**–**E**) Study day 0 SARS2-specific CD4^+^ T cell intracellular cytokine production of (**C**) GzmB, (**D**) TNF, or (**E**) IL-2 among surface CD40L^+^ cells. (**F**) Parts of a whole donut plot summary of intracellular cytokine (IFN-γ, GzmB, TNF, IL-2) production by SARS2-specific CD4^+^ T cells expressing 0 to 4 cytokines. (**G** and **H**) As in **A** and Supplemental Figure 2B but for SARS2-specific circulating Tfh cells (cTfh). Flow cytometry gates display frequency (%). Bars = geometric mean. Dotted lines = LOQ.

Both NP swab and peripheral blood samples were collected before treatment on study day 0. Acute adaptive immune responses were measured for all participants prior to treatment. Additionally, longitudinal responses were measured in the 49 placebo group participants after treatment on study days 7 and 28. We previously reported day 28 antibody, memory CD4^+^ T cell, and memory CD8^+^ T cell outcomes (20). Early (study day 0) SARS2-specific CD4^+^ T cell response magnitude and functionality were evaluated by multiple techniques and using multiple SARS2 antigens. Activation-induced marker (AIM) and hybrid AIM plus intracellular cytokine staining (AIM+ICS) T cell assays were employed (Figure 2, A and B, and Supplemental Figure 2A). SARS2 spike-specific (S-specific) and non-S-specific CD4^+^ T cells were measured by AIM assays following stimulation with S and non-S (CD4-RE) (28) peptide megapools (MPs). Two sets of AIM phenotyping surface protein marker pairs were utilized to assign AIM assay positivity (expression of surface OX40^+^41BB^+^, Figure 2A, and surface OX40^+^CD40L^+^, Supplemental Figure 2B). SARS2-specific CD4^+^ T cell frequencies were comparable by AIM based on OX40^+^41BB^+^ or OX40^+^CD40L^+^ surface marker expression (Figure 2A and Supplemental Figure 2B). Seventy-seven percent of individuals were positive for S-specific CD4^+^ T cells, and 79% of individuals were positive for non-S CD4^+^ T cells (Figure 2A). Combined responses to SARS2 S- and non-S epitopes were also quantified to evaluate total antiviral T cell responses per participant (Figure 2A and Supplemental Figure 2, B–G). Overall, substantial CD4^+^ T cell responses were observed.

SARS2-specific CD4^+^ T cells were phenotyped based on cytokine production by ICS following stimulation with the S and non-S MPs, including interferon-γ (IFN-γ), Granzyme B (GzmB), tumor necrosis factor (TNF), and interleukin-2 (IL-2) production, by CD40L^+^CD4^+^ T cells (Figure 2, B–F, and Supplemental Figure 2, H–J). IFN-γ was the most expressed cytokine at study day 0 (Figure 2B) and is known to be important in control of multiple viral infections in mouse models. Between 85% and 87% of individuals had SARS2-specific CD4^+^ T cell responses by AIM or IFN-γ ICS (combined S plus non-S responses, Figure 2, A and B). SARS2-specific CD4^+^ T cells were polyfunctional, with 69% producing at least 2 cytokines and 53% producing 3 or more cytokines (Figure 2, B–F). SARS2-specific circulating T follicular helper cells (cTfh, CXCR5^+^) were measured. Similar response rates were seen for SARS2-specific cTfh as for total SARS2-specific CD4^+^ T cells. SARS2-specific cTfh were observed in 79%–84% of participants’ study day 0 PBMCs (Figure 2, G and H). Overall, SARS2-specific CD4^+^ T cells with different functionalities and differentiation states were present.

**Figure 3:**
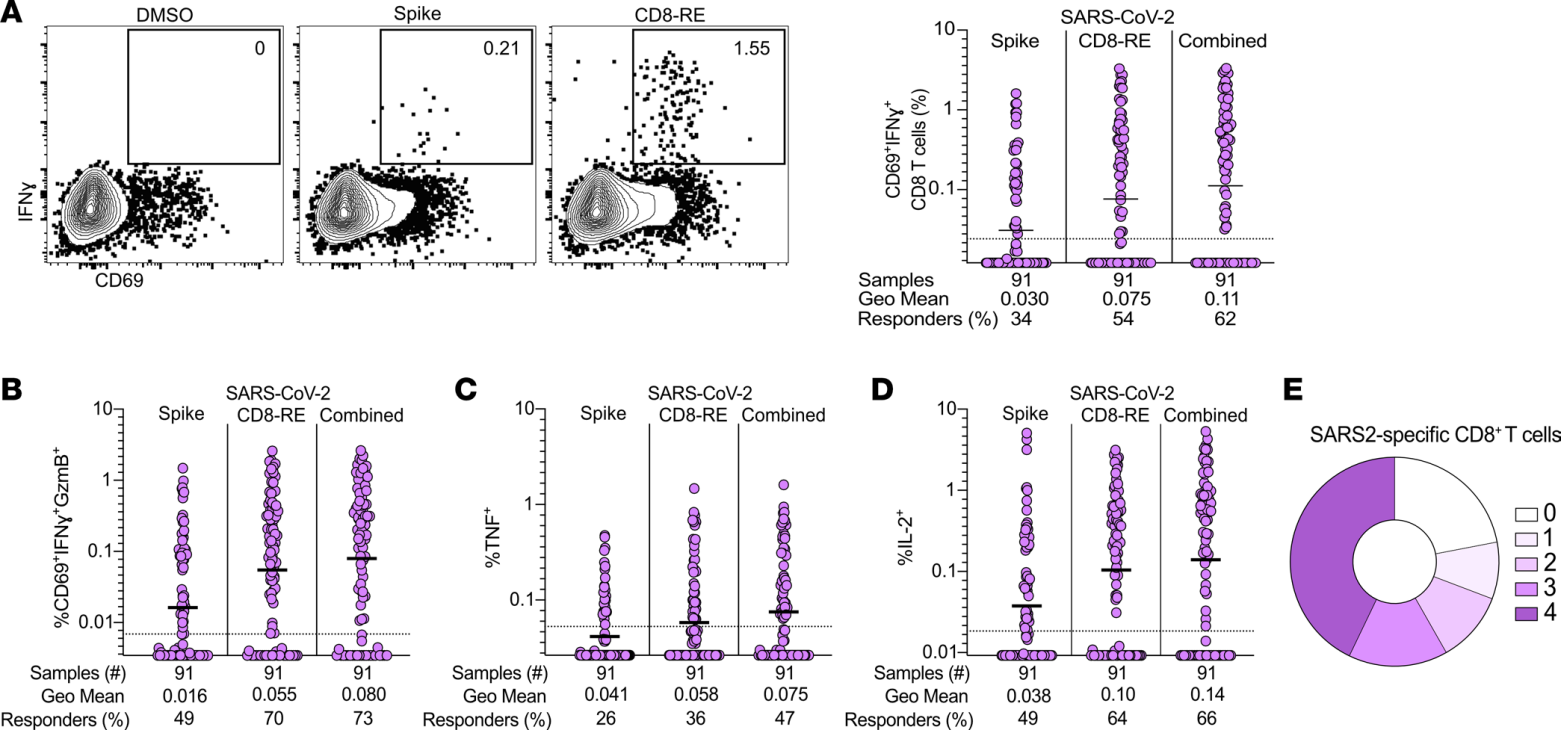
Antigen-specific CD8^+^ T cell responses to primary SARS2 infection and acute COVID-19. (**A**) Representative flow cytometry plots and frequency of SARS2-specific CD8^+^ T cells to DMSO (negative control), S, and CD8-RE MP stimulation conditions (Combined = sum of S + CD8-RE responses; see Methods for additional details) by IFN-γ^+^ ICS among CD69^+^ cells. (**B**–**D**) Production of (**B**) IFN-γ and GzmB, (**C**) TNF, and (**D**) IL-2. (**E**) Parts of a whole donut plot summary of intracellular cytokine (IFN-γ, GzmB, TNF, IL-2) production by SARS2-specific CD8^+^ T cells expressing 0 to 4 cytokines. Flow cytometry gates display frequency (%). Bars = geometric mean. Dotted lines = LOQ.

In parallel, study day 0 SARS2 S- and non-S-specific CD8^+^ T cell responses were evaluated by AIM (surface CD69^+^41BB^+^) and ICS following stimulation with SARS2 S and non-S (CD8-RE) (28) MPs (Supplemental Figure 3, A and D–F). Acute SARS2-specific CD8^+^ T cell responses were observed at study day 0 in 63% of individuals by AIM (Supplemental Figure 3B). By ICS, 62% of individuals were positive for an acute SARS2-specific CD8^+^ T cell response (surface CD69^+^ and intracellular IFN-γ^+^, Figure 3A). As was observed for SARS2-specific CD4^+^ T cells, IFN-γ was the cytokine most commonly produced by SARS2-specific CD8^+^ T cells (Figure 3A). CD8^+^ ICS was used as the primary quantification of SARS2-specific CD8^+^ T cells in downstream analyses given the similar detection rates of antigen-specific CD8^+^ T cells but lower background for the CD69^+^IFN-γ^+^CD8^+^ T cell assay (Figure 3A, LOQ) compared with CD8^+^ AIM (Supplemental Figure 3B, LOQ) (see Methods for additional details). As expected, CD8^+^ T cell responses did not vary on study day 0 when assessed by clinical trial group assignment (Supplemental Figure 3C). Most CD69^+^, IFN-γ–producing, SARS2-specific CD8^+^ T cells also produced GzmB (73%, Figure 3B and Supplemental Figure 3D). Similar to SARS2-specific CD4^+^ T cells, SARS2-specific CD8^+^ T cells were predominantly polyfunctional, with 69% producing at least 2 cytokines and 58% producing 3 or more cytokines (Figure 3, A–E). Overall, polyfunctional virus-specific CD4^+^ and CD8^+^ T cell responses were detected in a substantial proportion of individuals during acute primary SARS2 infection.

**Figure 4:**
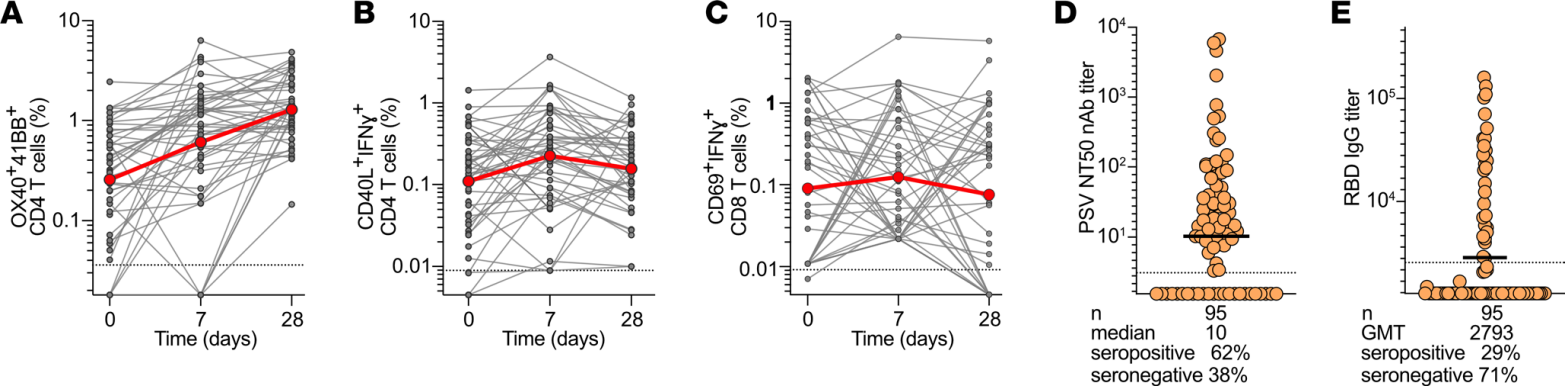
Longitudinal SARS2-specific T cell responses and Ab responses to primary SARS2 infection and acute COVID-19. (**A**–**C**) Longitudinal combined (S plus non-S) (**A**) CD4^+^ AIM, (**B**) CD4^+^ AIM+ICS, and (**C**) CD8^+^ IFN-γ^+^ T cell responses in placebo group (*n* = 49) participants at study days 0, 7, and 28. Red dots and lines represent median. Dotted line = LOQ. (**D**) Study day 0 pretreatment nAb titers for all participants. Dotted black line indicates LOD; seropositivity defined by values > LOD. Bar is median. nAb, neutralizing antibody. (**E**) Study day 0 pretreatment RBD IgG binding titers for all participants. Dotted black line indicates cutoff for seropositivity. Bar is geometric mean titer.

We examined the kinetics of T cell and antibody responses in the placebo group in more detail. Longitudinal assessment of SARS2-specific CD4^+^ and CD8^+^ T cell frequencies on study days 0 (Figures 2 and 3), 7 (Figure 4, A–C, and Supplemental Figure 4A), and 28 (Figure 4, A–C, and Supplemental Figure 4A) (20) showed that AIM^+^CD4^+^ T cell frequencies and the proportion of positive responders increased over time (Figure 4A and Supplemental Figure 4A). SARS2-specific IFN-γ^+^ CD4^+^ and CD8^+^ T cell frequencies remained stable from study days 0 to 28 (Figure 4, B and C), suggesting that antigen-specific, IFN-γ–producing T cells are formed early during primary infection and stably maintained at least through early convalescence. SARS2 serologic assessments included nAb titers against ancestral SARS2 by lentiviral pseudovirus neutralization assays (PSV NT50) and receptor binding domain (RBD) binding IgG titers measured on study day 0 (Figure 4, D and E) (21) for RBD IgG. Most individuals were found to have formed nAbs against SARS2 within 6 days PSO. On study day 0, 62% of participants had positive nAb titers (Figure 4D and Supplemental Figure 4B). As expected, nAb and RBD IgG titers increased between study days 0 and 7 (Supplemental Figure 4, D and E).

**Figure 5:**
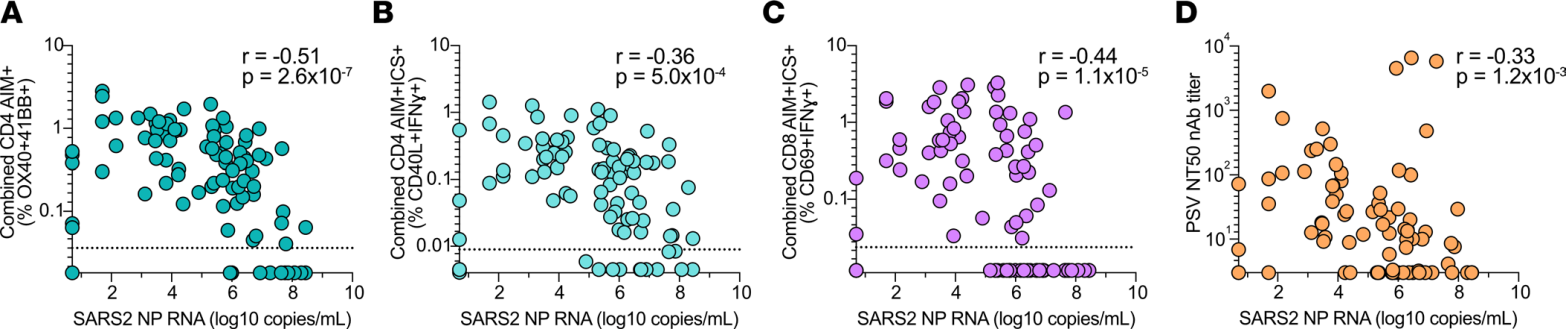
Correlative relationships between SARS2-specific adaptive immunity and upper airway viral RNA in acute COVID-19. (**A**–**D**) Relationships between study day 0 SARS2 NP RNA and combined day 0 SARS2-specific T cell responses by (**A**) CD4^+^AIM^+^, (**B**) CD4^+^IFN-γ^+^, (**C**) CD8^+^IFN-γ^+^, and (**D**) nAb titers.

To evaluate potential protective activities of early T cell responses, we examined associations between study day 0 NP viral RNA levels and the magnitude and diversity of SARS2-specific CD4^+^ and CD8^+^ T cell responses. The presence and magnitude of early SARS2-specific CD4^+^ T cell responses were associated with lower levels of SARS2 NP RNA based on all 3 main CD4^+^ T cell measurements of activation (OX40^+^41BB^+^ AIM Spearmanʼs *r* = –0.51, *P* = 2.6 × 10^–7^, Figure 5A; OX40^+^CD40L^+^ AIM *r* = –0.45, *P* = 9.3 × 10^–6^, Supplemental Figure 5A) and IFN-γ production (CD40L^+^IFN-γ^+^, *r* = –0.36, *P* = 5 × 10^–4^, Figure 5B). Early SARS2-specific CD8^+^ T cell response magnitude and IFN-γ production were also associated with lower levels of SARS2 NP RNA (IFN-γ^+^ *r* = –0.44, *P* = 1.1 × 10^–5^, Figure 5C; CD69^+^41BB^+^ AIM *r* = –0.39, *P* = 1.4 × 10^–4^, Supplemental Figure 5C). SARS2-specific T cell and SARS2 NP RNA associations were strongest when using combined S plus non-S T cell response measurements. Statistical associations were similar when S and non-S antigen-specific responses were evaluated separately though generally stronger for non-S than S-specific responses (Supplemental Figure 5, D–J). A positive correlation was observed between total SARS2-specific CD4^+^ and CD8^+^ T cell responses (Supplemental Figure 5K). A positive correlation was also observed between day 0 nAb and RBD IgG titers (Supplemental Figure 5L), as expected (48). Notably, SARS2 nAb titers measured at study day 0 were also associated with lower levels of SARS2 NP RNA, but the association with nAb was weaker than the association with either day 0 SARS2-specific CD4^+^ or CD8^+^ T cells (*r* = –0.33, *P* = 1.2 × 10^–3^, Figure 5D).

Given the biological dependence of the antibody response on CD4^+^ T cell help mediated by Tfh (50, 51), some statistical association between antibodies and viral load would always be expected, even if, hypothetically, viral control were exclusively mediated by T cells. Early S-specific cTfh had relatively modest associations with SARS2 NP RNA that were weaker than total S-specific CD4^+^ T cell AIM response associations but similar to nAb titer associations (Spearman’s *r* = –0.37 to 0.39, *P* = 1.6 × 10^–4^ to 2.8 × 10^–4^, Supplemental Figure 5, I and J). Analysis of covariance (ANCOVA) testing was performed to better evaluate if the putative protective relationships observed between early SARS2-specific CD4^+^ T cell responses and SARS2 NP RNA remained after accounting for antibodies, based on day 0 nAb serostatus. Associations between total SARS2-specific CD4^+^ T cell or SARS2-specific cTfh responses and lower SARS2 NP RNA did not show a dependence on SARS2 nAb seropositivity (Supplemental Figure 5, M–O). SARS2-specific CD8^+^ T cell associations with lower SARS2 NP RNA were also independent of nAb seropositivity (Supplemental Figure 5M). Thus, early SARS2-specific CD4^+^ and CD8^+^ T cell responses were specifically associated with reduced upper airway SARS2 viral loads in unvaccinated, nonhospitalized cases of acute COVID-19.

Examining SARS2 antigen-specific CD4^+^ and CD8^+^ cytokine production and associations with viral NP RNA at study day 0 revealed that in addition to IFN-γ production, production of the Th1 cytokines GzmB, IL-2, and TNF similarly correlated with lower NP viral RNA levels (Supplemental Figure 6, A–F). These associations were similar for cytokine-producing CD4^+^ and CD8^+^ T cells recognizing both S and non-S epitopes. For SARS2-specific CD4^+^ T cells, associations were similar regardless of which cytokine the cells were producing, but for SARS2-specific CD8^+^ T cells, production of both IFN-γ and GzmB was most strongly associated with decreased SARS2 NP RNA (Supplemental Figure 6D). Thus, diverse and polyfunctional SARS2-specific CD4^+^ and CD8^+^ T cell responses, including CD4^+^ and CD8^+^ T cells with profiles of cytotoxicity, may play a role in viral clearance during acute SARS-CoV-2 primary infection.

Participants in the study enrolled at a range of days PSO, which provides richness to the dataset but raised the possibility that the immune response associations with reduced NP viral RNA levels might only be surrogates for time from infection. To test this possibility, an analysis was performed to try to account for differences in time from symptom onset to study entry, using simple log-linear regression models of the raw data to extrapolate SARS2-specific immune response and NP RNA data to day 6 PSO (the median number of days PSO for all participants) (Supplemental Figure 7, A–E, see Methods). If the original SARS2-specific T cell associations with reduction of SARS2 NP RNA levels were only surrogates of time from infection (measured as days PSO), T cell associations should be lost upon adjustment for days PSO. If instead the T cell associations reflected a functional relationship, statistical associations between T cell responses and lower viral RNA would be expected to remain after adjustment for days PSO. Although weaker, the relationships between CD4^+^ and CD8^+^ T cell responses and viral NP RNA levels adjusted to 6 days PSO remained statistically significant (CD4^+^ AIM Spearman’s *r* = –0.39, *P* = 1.9 × 10^–4^, Supplemental Figure 7F; CD4^+^IFN-γ^+^ *r* = –0.35, *P* = 1.0 × 10^–3^, Supplemental Figure 7G; CD8^+^IFN-γ^+^ *r* = –0.31, *P* = 4.4 × 10^–3^, Supplemental Figure 7H), consistent with a functional role for T cells in control of viral NP RNA levels. Associations between nAb titers and viral NP RNA levels also remained after adjustment (*r* = –0.32, *P* = 3.0 × 10^–3^, Supplemental Figure 7I). Together, these data are evidence of functional relationships in humans between the development of early antiviral CD4^+^ and CD8^+^ T cell responses, and antibodies, and control of acute SARS2 infection in the upper airway.

In summary, this study provides insights into the relationship between acute adaptive immune responses to SARS2 and viral control. It has been hypothesized that SARS2-specific antiviral T cell responses may contribute to protection from symptomatic and severe disease by reducing viral burden and enhancing the speed of viral clearance; however, few human studies have directly measured the role of cellular immunity on viral control of primary infection (36, 39). Here, using samples from 95 individuals who participated in a randomized, controlled clinical trial, the largest virus-specific acute immune response data set of its kind was generated for adults up to 86 years of age, including many individuals at risk for progression to severe COVID-19.

## Discussion

The data demonstrate that (a) SARS2-specific CD4^+^ and CD8^+^ T cells could be detected in the majority of adults with outpatient COVID-19 within days from symptom onset, (b) the T cells were polyfunctional, (c) stronger early SARS2-specific CD4^+^ T cell responses were associated with lower viral RNA levels in the upper airway, (d) stronger SARS2-specific CD8^+^ T cell responses were associated with lower viral RNA levels in the upper airway, (e) the associations of T cell responses with lower viral RNA were independent of nAb responses, and (f) the associations remained significant after accounting for time from COVID-19 symptom onset. The conclusions from these analyses of T cell associations with viral control were reproducible by multiple measurements of CD4^+^ T cell and CD8^+^ T cell functionalities and SARS2 antigen specificities. Overall, these findings suggest that SARS2-specific T cells play a role in protective immunity to SARS2 during acute infection, which is consistent with previously published studies.

In a smaller study (*n* = 25 acute infections sampled), Eser et al. found a strong association between nucleocapsid-specific (N-specific) IFN-γ^+^CD4^+^ T cells and lower viral loads (39). Those results are consistent with the CD4^+^ T cell findings here for non-S antigen pools, which included N-specific peptides. Additionally, we found that T cell responses to S also associated with reduced viral loads, though somewhat less than for non-S responses. That Eser et al. observed less of an association for N-specific IFN-γ^+^CD8^+^ T cell responses and lower viral loads was perhaps because the study sample size was limited and fewer participants developed early circulating N-specific IFN-γ^+^ CD8^+^ than CD4^+^ T cell responses (39).

In a human controlled SARS2 infection study of healthy young adults (*n* = 18 infected, mean age of 22 years old) with a limited infectious dose (53% of individuals were infected), total CD38^+^Ki67^+^CD8^+^ T cells in peripheral blood had the strongest association with accelerated viral clearance (36). Those data are consistent with the CD8^+^ T cell findings here regarding early SARS2-specific CD8^+^ T cell activation being associated with reduced viral NP RNA. A strength of that study was the precisely defined time of infection and longitudinal tracking of viral loads and T cell responses in blood based on markers. Several factors distinguish the study reported herein. Early SARS2 S- and non-S-specific CD8^+^ T cells both correlated with viral clearance, including when quantified by 2 T cell assays (AIM and/or IFN-γ production). Additionally, early SARS2 total and S- and non-S-specific CD4^+^ T SARS2-specific responses also correlated with lower viral loads, including SARS2-specific cTfh. The cohort size here is substantially larger (95 vs. 18) and presumably includes people infected by a range of viral exposure doses. The virus-specific T cell assays herein measured CD4^+^ and CD8^+^ T cell epitopes spanning the S protein and non-S antigens, including both structural and nonstructural proteins, and we were able to detect early circulating SARS2-specific T cells. Given that nonstructural proteins are expressed earlier in infection and are better conserved across human coronaviruses, it is possible that the inclusion of nonstructural protein epitopes in our peptide megapools allows for earlier and more comprehensive detection of both cross-reactive and SARS2-specific circulating T cell responses than peptide pools containing only SARS2 structural protein epitopes (38, 52, 53). Additionally, the data herein demonstrate that the early SARS2-specific CD4^+^ and CD8^+^ T cells were polyfunctional, not only defined by IFN-γ production (36, 39). Last, in the controlled infection study (36), all participants were young healthy adults (mean age 22 years old), while the participants herein were from a broad age range (18–86 years old), and half had comorbid conditions considered high risk for development of severe COVID-19. Thus, the data herein represent a broad age spectrum and are particularly relevant for those age groups and demographics most at risk for worse outcomes due to COVID-19, such as hospitalization or death.

This study in no way excludes a complementary role for antibodies in control of SARS2 in unvaccinated individuals. Our study has limitations. It is intrinsically challenging to disentangle roles of T cells from roles of antibodies in combating SARS2 in humans (1–3). This study provides evidence that SARS2-specific T cell responses exhibit selective correlates with viral clearance, but it is entirely plausible that coordinated adaptive immunity of T cells and antibodies are important in protection (14, 16, 24). Additionally, adaptive immune responses at the site of initial infection were not directly assessed in this study and may not be adequately captured by evaluation of circulating immune cells and antibodies from peripheral blood samples. Thus, a role for local, upper airway cellular and humoral immunity in protection from SARS2 cannot be excluded (16, 36, 55–57). The study was also underpowered to distinguish the myriad roles that different CD4^+^ and CD8^+^ T cell subsets may individually play in viral clearance or to determine whether SARS2-specific CD4^+^ and CD8^+^ T cell responses are independent. Associations between early SARS2-specific CD4^+^ and CD8^+^ T cell responses and NP viral RNA were independent of both nAb serostatus and time from SARS2 infection using relatively simple statistical tests. While this is in agreement with other studies indicating roles for T cells in protection against SARS2 infection (24, 36, 39), the use of more sophisticated mathematical modeling may provide additional insights. Last, our findings focused on primary infection with ancestral SARS2 and may not be reflective of all individuals with acute COVID-19, including primary infection by SARS2 variants or SARS2 breakthrough infections.

While T cell kinetics and phenotypes in response to SARS2 infection and COVID-19 vaccination may not be identical and are shaped by exposure history (28, 40, 58), the cumulative data now indicate that SARS2-specific T cells play a protective role in both primary SARS2 infection and breakthrough infections. It has been observed that in COVID-19–vaccinated individuals who experience breakthrough infections, recall responses include rapid activation of memory S-specific CD4^+^ and CD8^+^ T cells within the first week, which precede humoral responses, and that S-specific CD8^+^ T cells are associated with accelerated viral clearance. Given the overall findings in the field, next-generation SARS2 or pan-sarbecovirus vaccines may benefit from eliciting enhanced T cell responses and from clinical trials that assess both baseline and vaccination-induced SARS2-specific CD4^+^ and CD8^+^ T cell responses and functionality.

## Methods

### Sex as a biological variable.

Our study examined similar numbers of males and females as SARS-CoV-2 infects both men and women (Table 1). Similar findings are reported for both males and females.

### Study population and trial.

ACTIV-2/A5401 was a multicenter phase II/III randomized controlled trial designed to evaluate the safety and antiviral and clinical efficacy of therapeutics for acute COVID-19 in nonhospitalized adults. Individuals in this study represent a subset of participants from ACTIV-2/A5401 who received a single intravenous dose of 700 mg of bamlanivimab or normal saline placebo (comparator control group) and had available clinical data (e.g., demographics, days PSO, risk for severe COVID-19; Table 1) (21, 47) and blood samples for immunologic testing. Samples from nearly half of the overall bamlanivimab 700 mg and comparator placebo groups were available for this study. Approximately equal numbers of participants in the placebo group for this study were considered high risk versus low risk for progression to severe COVID-19 (Table 1). This was also true for the bamlanivimab treatment group (Table 1). Participants were from the United States and enrolled in ACTIV-2/A5401 between October and November 2020. At study entry, COVID-19 vaccines were not available to the population in the United States, and SARS2 infections could be attributed to ancestral SARS2 virus. The participants in this study were comparable at study entry across treatment groups (Table 1) (21, 47). Inclusion criteria for ACTIV-2/A5401 included adults 18 years or older with no more than 10 days of COVID-19 symptoms and documented SARS2 infection by FDA-authorized antigen or molecular testing within 7 days prior to study entry. Participants were assigned to the bamlanivimab or placebo groups at a 1:1 ratio, and randomization was stratified by time from symptom onset (< or ≥ 5 days PSO) and risk of progression to severe COVID-19 based on age (< or ≥ 55 years old) and the presence or absence of predefined comorbid medical conditions (e.g., body mass index > 35 kg/m^2^, hypertension, diabetes) (46). Additional information for ACTIV-2/A5401 is available at ClinicalTrials.gov (Identifier: NCT04518410) and in the primary outcomes manuscript (46). Three individuals were noted to have high NP viral loads despite high nAb titers at study day 0 (Figure 5D). Notably, 1 of these 3 individuals was in the placebo group. It was subsequently reconfirmed that blood was drawn for all participants prior to bamlanivimab or placebo infusion and that none of the placebo group participants received bamlanivimab or any other active drug treatment for COVID-19.

Partial data for day 0 RBD IgG titers by group, similar to the data shown in Supplemental Figure 4C but with smaller sample sizes and without using a strict MFI-based cutoff, were previously published (20). Although combined day 28 AIM and AIM+ICS data were published to show T cell response rates between the treatment and placebo groups (20), they were not presented as longitudinal data (as shown in Figure 4, A–C, and Supplemental Figure 4A). Day 0 and 7 T cell data have not been previously published.

### SARS2 viral RNA.

Samples were collected, processed, and analyzed as previously reported (46). In brief, NP swab samples were collected by ACTIV-2/A5401 trial staff at designated study sites on assigned study days (days 0, 7, 14, and 28) using standardized swabs and validated collection and storage procedures. NP swabs were frozen upon collection and stored at –80°C (–65°C to –95°C) until shipment on dry ice to a central laboratory (University of Washington) for extraction, amplification, and quantitative detection testing using validated, previously published methods using the Abbott *m*2000sp/rt system with a validated controls and standards for correlation with cycle threshold and viral load. The LOD was 1.4 log_10_ copies/mL, lower LOQ was 2 log_10_ copies/mL, and upper limit of quantification (ULOQ) was 7 log_10_ copies/mL for this assay. For samples with viral RNA levels above the ULOQ, samples were diluted, and the assay was repeated to obtain a quantitative value.

### PBMCs and viability-based quality control.

Peripheral blood was collected by ACTIV-2/A5401 trial staff at designated study sites on assigned study days. Serum and PBMCs for immunologic testing were isolated from whole blood using standard operating procedures. PBMCs were cryopreserved and stored in liquid nitrogen until ready for use, then thawed at 37°C, resuspended in warm complete RPMI medium with 5% human AB serum (Gemini Bioproducts) and benzonase, and centrifuged at 500*g* to remove cryopreservation medium. Following washing, cell counts were performed, and viability was assessed using the Muse Count & Viability Kit (Muse Cell Analyzer; Luminex). A PBMC viability threshold was set at ≥75% for all samples for inclusion in data analysis. PBMCs with viability below this threshold failed to appropriately respond to control stimuli. Four of 95 PBMC samples failed to meet the viability cutoff and were excluded from analyses. PBMCs were resuspended to achieve a final concentration of 100,000 PBMCs/100 μL for plating in 96-well format for T cell assays. Cryopreserved convalescent COVID-19 donor PBMCs from known positive responders obtained from the Sette lab under a protocol (VD-214) approved by La Jolla Institute for Immunology (LJI) Institutional Review Board (IRB) served as batch controls across T cell assays. These PBMCs were handled in the same fashion as ACTIV-2/A5401 PBMC samples. Additional quality control metrics were applied, as described below for the individual T cell assays.

### AIM assay.

As previously described (20), PBMCs were cultured in 96-well, U-bottom plates for 24 hours at 37°C in an incubator with 5% CO_2_ in the presence of a single stimulus per well: a negative control (equimolar amount of DMSO vehicle), a positive control (Staphylococcal enterotoxin B [SEB] at 1 μg/mL), or a single SARS2 MP (1 μg/mL per MP) (19, 28, 59) containing S (19, 59) or paired SARS2 non-S epitope MP (CD4-RE or CD8-RE dominant and subdominant MPs) (28). The S MP was composed of overlapping peptides spanning the full-length ancestral S protein. The CD4-RE MPs (non-S) consisted of experimentally validated and optimized class II epitopes from the remainder of the SARS2 proteome (outside the S open reading frame, ORF). CD8^+^ AIM responses were calculated using the AIM+ICS assay, as PBMC counts were limited and did not allow for CD8-RE MP wells to be plated for both AIM and AIM+ICS assays (see *Hybrid AIM+ICS* section below for additional experimental details).

PBMCs were plated at 1 × 10^6^ PBMCs per MP stimulation well and between 0.5 × 10^6^ and 1 × 10^6^ PBMCs per control well; negative controls were plated in duplicate. Prior to stimulation, PBMCs were incubated at 37°C for 15 minutes with 0.5 μg/mL anti-human CD40 blocking antibody (Miltenyi Biotec) per well. Chemokine receptor antibodies were also added to each well on day 1 (reviewed in ref. 20; see Supplemental Table 1 for antibodies used in the AIM assay). After a 24-hour incubation, the plates were centrifuged at 500*g*, and cells were washed with PBS, stained with LIVE/DEAD Fixable Blue (Invitrogen) 1:1,000 in PBS with Fc block (5 μL/sample; BD Biosciences [BD]) for 15 minutes at room temperature, washed with FACS buffer (3% FBS in Dulbeccoʼs PBS without calcium or magnesium), surface stained (reviewed in ref. 20 for surface staining panel; 30 minutes at 4°C), fixed with BD Cytofix Fixation Buffer (4°C for 20 minutes), and stored at 4°C (up to overnight) until flow cytometric analysis was performed using a 5-laser Aurora (Cytek Biosciences). Flow cytometric data were acquired separately for each sample and stimulation condition; no samples were pooled. Analysis was performed using FlowJo (BD), and AIM^+^ gates were drawn based on MP-stimulated responses relative to negative (DMSO) and positive (SEB) controls. T cell assays on 2 SARS2 convalescent control PBMC samples were run with each set of experimental PBMC samples to account for batch effects; aliquots from the same PBMC controls were used across batches. A single gating strategy was applied to all samples across all batches unless there was a clear batch-specific effect to justify modifying the gates for a specific batch of samples. Our standard acceptable background (% AIM^+^ T cells) for DMSO gates is 0.1. DMSO gates were set to target average background values less than or equal to 0.1 using the same flow cytometry gating strategy for all samples across all batches. CD4^+^ and CD8^+^ T cell responses to MP versus DMSO stimulation are shown in Supplemental Figure 2, K–M, and Supplemental Figure 3, G and H. Higher background was allowed for CD8^+^ AIM (CD69^+^41BB^+^) assays (Supplemental Figure 3G) based on higher background observed for CD8^+^ AIM both here and in prior studies (20). ICS (CD69^+^IFN-γ^+^) was preferentially used as the main readout for SARS2-specific CD8^+^ T cell responses given lower background (Supplemental Figure 3H) with comparable response rates to CD8^+^ AIM.

PBMC quality was evaluated by measuring the median response to SEB for all samples, and samples with responses less than 50% of the overall median SEB response were excluded from downstream analyses. Two DMSO negative-control well replicates were run for each sample, and the average of the individual DMSO wells for each sample was calculated. T cell responses were verified by stimulation index (SI) as a quality control. SI was calculated for each sample as the fold-change in responses to MP stimulation versus the average response to DMSO for the same participant (as shown in Supplemental Figure 2, N–P, and Supplemental Figure 3, I and J). An SI cutoff of 2 was used for determining AIM^+^CD4^+^ T cell responses and 3 for AIM^+^CD8^+^ T cell responses. The higher SI cutoff for CD8^+^ T cell responses was applied based on higher background for CD8^+^ AIM assays. When a nonzero DMSO value was needed for calculating the SI, the minimum DMSO signal was set to 0.005%. For each MP-stimulated sample, the average DMSO value was subtracted from the MP response to calculate the background-subtracted MP response. Background-subtracted S and non-S values are shown for all figures. Combined AIM responses were calculated as the mathematical sum of the background-subtracted responses to individual (S plus non-S) MPs. In the case of negative AIM responses to both S and non-S megapools, or summative responses that were less than the lower LOQ for the assay, as described below, the combined AIM response was also considered negative. The geometric mean of all DMSO control wells for all samples for each assay was calculated to determine the LOQ for the assay. Positive responders were defined as those with background-subtracted responses greater than the LOQ. The baseline for the *y* axis was set to 0.5 × LOQ. All nonresponders were set to this baseline.

### Hybrid AIM+ICS.

AIM+ICS assays were also performed as previously described (20). For CD4^+^ T cells, intracellular cytokine expression was measured in conjunction with surface CD40L. For CD8^+^ T cells, ICS was measured in conjunction with surface CD69. PBMCs were thawed and plated in parallel and as for AIM assays but with the addition of wells for CD8-RE MPs (paired dominant and nondominant epitope pools) (28). The CD8-RE pools (non-S) consisted of optimized, experimentally validated class I epitopes from the remainder of the SARS2 proteome (outside the S ORF) (28). Cells were incubated with anti-CD40 blocking antibody as for AIM assays, but no chemokine receptor antibodies were added on day 1, except for CXCR5 for identification of cTfh (reviewed in ref. 20 for antibodies used). After 20–22 hours, PMA (0.05 μg/mL) and ionomycin (0.25 μg/mL) were added to the positive (ICS) control wells. Two hours later, 0.25 μL/well of GolgiStop (BD) and GolgiPlug (BD) and the AIM marker antibodies (20) were added to all samples, and the plates were incubated for another 4 hours at 37°C (in a 5% CO_2_ incubator). Cells were then washed, surface stained for 30 minutes at 4°C, fixed, and washed using Cytofix/Cytoperm (BD) per the manufacturer’s protocol. ICS was then performed using antibodies diluted in Perm/Wash Buffer (BD) for 30 minutes at 4°C. Cells were washed with Stain Buffer with FCS (BD) and stored in this buffer at 4°C until flow cytometric analysis was performed using a Cytek Aurora. Flow cytometric data were acquired separately for each sample and stimulation condition.

Gating and quality control evaluations with SEB responses and SI calculations were performed as described above for AIM assays. PMA + ionomycin served as an additional control for cytokine production (ICS). For each MP-stimulated sample, the average DMSO value was subtracted from the MP response to calculate the background-subtracted MP response. Background-subtracted values are shown for all figures unless indicated. Combined responses were calculated as the sum of the background-subtracted responses to individual MPs (S and CD4-RE for CD4^+^ T cells, S and CD8-RE for CD8^+^ T cells). In the case of negative ICS responses to both S and non-S MPs, the combined response was also considered negative. The LOQ and baseline were calculated and plotted as described above for AIM assays.

### Binding antibody titers.

Serum SARS2-specific binding antibody assays were performed using the Bio-Plex Pro Human SARS2 IgG 4-Plex Panel serology assay (Bio-Rad 12014634) according to the manufacturer’s protocol, as previously described (20). MFI-based cutoffs for seropositivity are indicated in the figures.

### Pseudovirus neutralizing antibody titers.

Half-maximal nAb titers (NT50) were generated using a lentiviral PSV assay, as previously described (59). Briefly, PSV was generated in HEK293T cells cotransfected with SARS2 S expression and pNL4–3.luc.R-E-mCherry-luciferase reporter packaging plasmids. The cells were from ATCC, and plasmid was generated using reagents from Addgene and the NIH HIV Reagent Program (59). Serum was isolated from the peripheral blood of study participants using standard protocols, frozen, and shipped to the testing site without thawing until ready for use. The serum was then serially diluted and incubated with SARS2 PSV, then HEK293T cells stably expressing human angiotensin-converting enzyme 2. Serum dilutions were run in triplicate. The percentage inhibition was calculated using relative light units emitted by the reporter at each dilution and plotted versus the log serum dilution. The NT50 was then derived by nonlinear regression. The LOD for the assay was defined by the lowest dilution tested (1:3) as indicated in Figure 4D and Supplemental Figure 4B.

### Statistics.

Statistical analyses were performed in GraphPad Prism 10 and Microsoft Excel. Comparisons of SARS2-specific T cell response magnitudes and antibody titers between treatment and placebo groups for equivalent conditions were determined by Mann-Whitney *U* tests. Fisher’s exact tests were used to compare participant characteristics (Table 1) and to compare SARS2-specific T cell positive and negative response rates between treatment and control groups. Kruskal-Wallis nonparametric tests with post hoc Dunn’s multiple-comparison tests were used for assessing T cell responses across more than 1 stimulation condition or cell type. Relationships between SARS2 NP RNA levels and immune responses were assessed using nonparametric Spearman’s correlations. One-way ANCOVA was performed on unadjusted viral RNA and immune response data to determine if the relationships between SARS2 NP RNA levels and SARS2-specific T cell responses at study day 0 were dependent on SARS2 nAb serostatus (Supplemental Figure 5, M–O).

Individual variation in days from symptom onset to study entry (day 0) was accounted for using a log-linear regression model (Supplemental Figure 7, A–E). Adjusted SARS2 NP RNA levels (Supplemental Figure 7A) and adaptive immune responses (log_10_-transformed CD4^+^ T cell responses, CD8^+^ T cell responses, and nAb titers; Supplemental Figure 7, B–E) at 6 days PSO (the median time from symptom onset at study entry) were calculated for all participants using the linear regression models shown in Supplemental Figure 7, A–E, to adjust the data shown in Figure 5, A–D. Data from 5 individuals were excluded from this analysis as outliers, as these individuals had undetectable viral RNA on study day 0 but were reportedly 4–5 days PSO as of study entry. This included 4 values from individuals who consistently tested negative for viral RNA (from both nasal and NP swabs) on all study days, and a value for 1 individual who tested negative for viral NP RNA on study day 0 but who subsequently tested positive for viral NP RNA on study days 3, 7, and 14. The adjusted NP viral RNA and adaptive immunity data were plotted and analyzed using nonparametric Spearman’s correlations to be able to compare the original (unadjusted) data (Figure 5, A–D) with the adjusted data (Supplemental Figure 7, F–I) using the same correlation method.

For all analyses, a 2-sided 5% type I error rate was used, without adjustment for multiple comparisons, except as noted above. Additional details can be found in the Results, figures, and corresponding legends.

### Study approval.

The ACTIV-2/A5401 clinical trial protocol (ClinicalTrials.gov Identifier: NCT04518410) was approved by a central IRB: Advarra, Columbia, Maryland, USA (Pro00045266). The LJI IRB provided additional oversight for this study. All individuals enrolled in ACTIV-2/A5401 provided written informed consent for participation.

### Data availability.

The authors confirm that the source data underlying the findings are fully available. Due to ethical restrictions, additional ACTIV-2/A5401 clinical trial study data beyond what are presented in this manuscript and supplement are available upon request from the AIDS Clinical Trials Group (ACTG) and with the written agreement of ACTG and the manufacturer of the investigational product. Completion of a data use agreement may be required. Values for all data points in graphs are reported in the Supporting Data Values file.

## Author contributions

SIR, DMS, and SC conceptualized the study. SIR, PGL, FF, and AH investigated. SIR and SC performed formal analysis for the study. DMS recruited patients and provided samples. DMS, KWC, MDH, JJE, JC, DW, ESD, JR, and CM were involved in clinical trial design and oversight. UMP and AH provided data and resources for serologic analyses. A Greninger provided data and resources for viral RNA analyses. PK, A Grifoni, DW, and AS provided additional material resources. SIR, FF, BP, DMS, and SC wrote the manuscript. DMS and SC supervised the study.

## Supplementary Material

Supplemental data

Supporting data values

## Figures and Tables

**Table 1:**
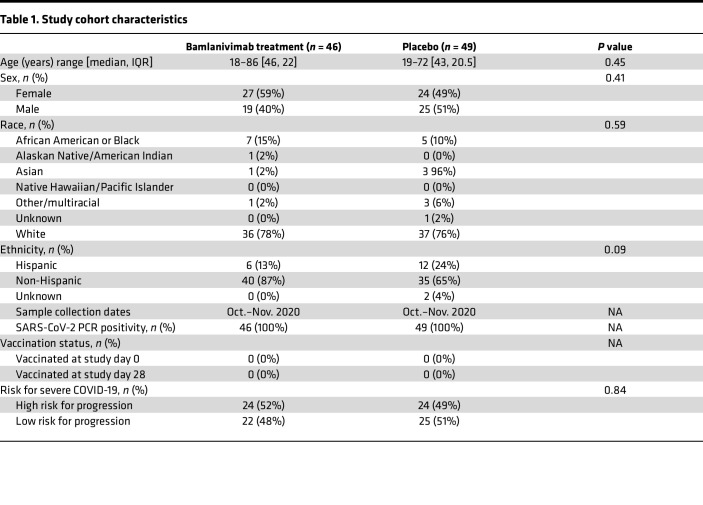
Study cohort characteristics

## Version 1 12/20/2024

Electronic publication
