# Supplementary material for: Early antiviral CD4+ and CD8+ T cells are associated with upper airway clearance of SARS-CoV-2
Source: JCI Insight. 2024 Dec 20;9(24):e186078. doi: 10.1172/jci.insight.186078 (PMC11665554; doi:10.1172/jci.insight.186078)
Supplement: Supplemental data [file jciinsight-9-186078-s083.pdf]

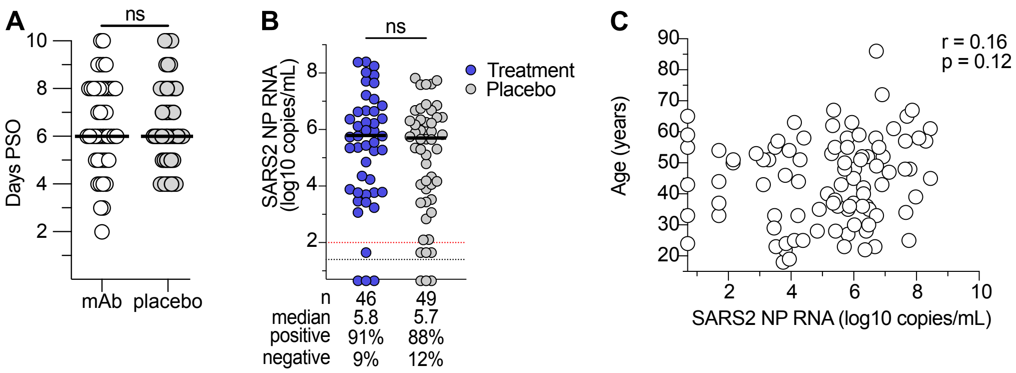

Figure S1. Days from symptom onset to study entry and SARS2 NP RNA at study entry by clinical trial group.
A. Days PSO at study day 0 for bamlanivimab (mAb; n = 46) and placebo group participants (n = 49); line = median (day 6). **B.** SARS2 NP RNA at day 0 by treatment group (Treatment = bamlanivimab). **C.** Relationship between day 0 SARS2 NP RNA and participant age. Lines and bars as in **Fig. 1B-C**. ns = not significant by Mann-Whitney test. **Related to Figure 1.**

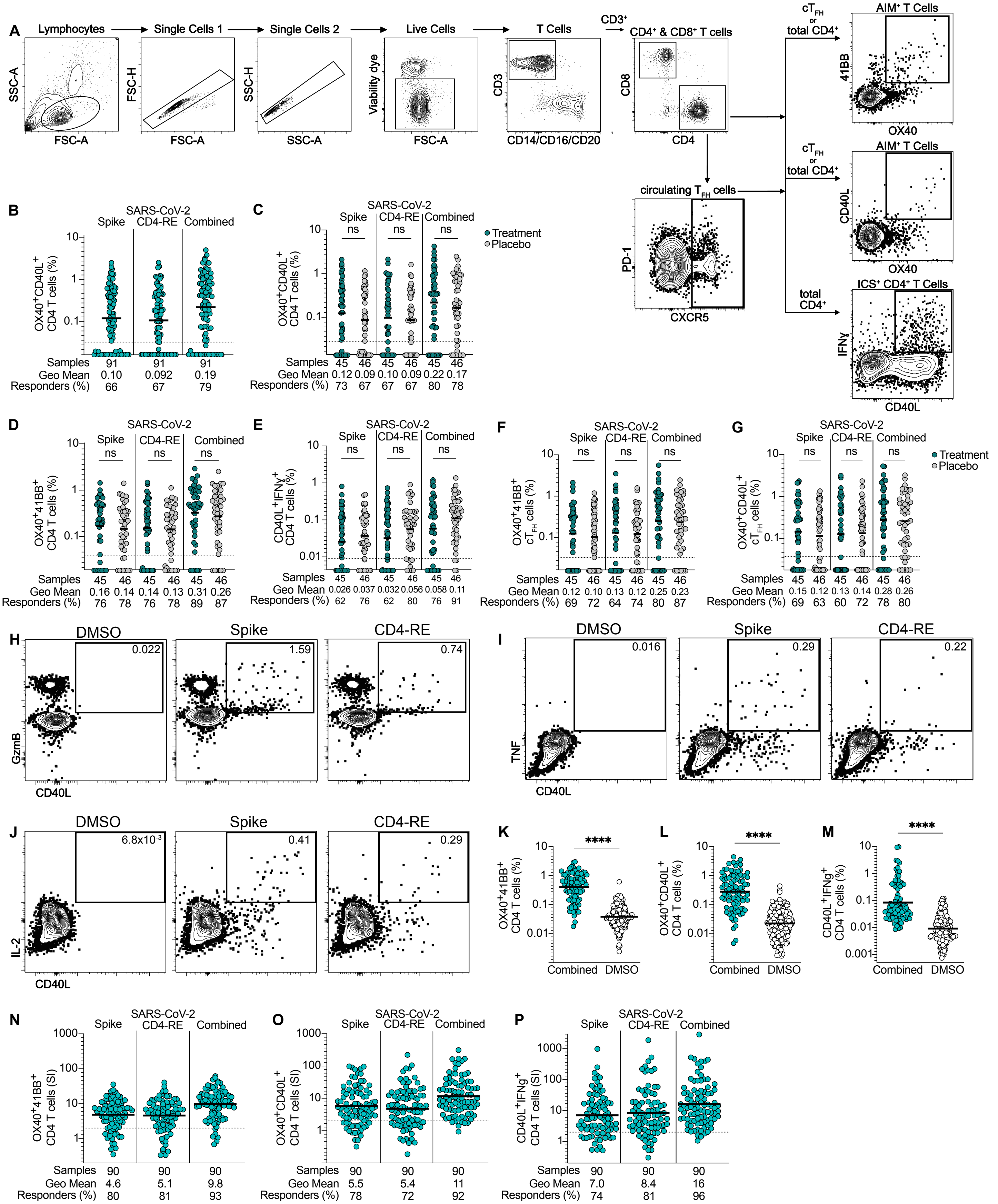
A
Lymphocytes
Single Cells 1
Single Cells 2
Live Cells
T Cells
CD3+
CD4+ & CD8+ T cells
SSC-A
FSC-A
FSC-H
SSC-H
Viability dye
CD3
CD14/CD16/CD20
CD8
CD4
cTFH or total CD4+
AIM+ T Cells
41BB
OX40
CD40L
circulating TFH cells
PD-1
CXCR5
total CD4+
ICS+ CD4+ T Cells
IFNγ
B
SARS-CoV-2
Spike
CD4-RE
Combined
OX40+CD40L+ CD4 T cells (%)
Samples 91 91 91
Geo Mean 0.10 0.092 0.19
Responders (%) 66 67 79
C
SARS-CoV-2
Spike ns
CD4-RE ns
Combined ns
Treatment
Placebo
OX40+CD40L+ CD4 T cells (%)
Samples 45 46 45 46 45 46
Geo Mean 0.12 0.09 0.10 0.09 0.22 0.17
Responders (%) 73 67 67 67 80 78
D
SARS-CoV-2
OX40+41BB+ CD4 T cells (%)
Samples 45 46 45 46 45 46
Geo Mean 0.16 0.14 0.14 0.13 0.31 0.26
Responders (%) 76 78 76 78 89 87
E
SARS-CoV-2
CD40L+IFNγ+ CD4 T cells (%)
Samples 45 46 45 46 45 46
Geo Mean 0.026 0.037 0.032 0.056 0.058 0.11
Responders (%) 62 76 62 80 76 91
F
SARS-CoV-2
OX40+41BB+ cTFH cells (%)
Samples 45 46 45 46 45 46
Geo Mean 0.12 0.10 0.13 0.12 0.25 0.23
Responders (%) 69 72 64 74 80 87
G
SARS-CoV-2
OX40+CD40L+ cTFH cells (%)
Samples 45 46 45 46 45 46
Geo Mean 0.15 0.12 0.13 0.14 0.28 0.26
Responders (%) 69 63 60 72 78 80
Treatment
Placebo
H
DMSO 0.022
Spike 1.59
CD4-RE 0.74
GzmB
CD40L
I
DMSO 0.016
Spike 0.29
CD4-RE 0.22
TNF
CD40L
J
DMSO 6.8x10-3
Spike 0.41
CD4-RE 0.29
IL-2
CD40L
K
****
OX40+41BB+ CD4 T cells (%)
Combined DMSO
L
****
OX40+CD40L+ CD4 T cells (%)
Combined DMSO
M
****
CD40L+IFNg+ CD4 T cells (%)
Combined DMSO
N
SARS-CoV-2
Spike
CD4-RE
Combined
OX40+41BB+ CD4 T cells (SI)
Samples 90 90 90
Geo Mean 4.6 5.1 9.8
Responders (%) 80 81 93
O
SARS-CoV-2
OX40+CD40L+ CD4 T cells (SI)
Samples 90 90 90
Geo Mean 5.5 5.4 11
Responders (%) 78 72 92
P
SARS-CoV-2
CD40L+IFNg+ CD4 T cells (SI)
Samples 90 90 90
Geo Mean 7.0 8.4 16
Responders (%) 74 81 96

Figure S2. Antigen-specific CD4 T cell responses to primary SARS2 infection and acute COVID-19.

A. Gating strategy and representative flow cytometry gating for CD4 AIM and AIM+ICS assay readouts using IFNγ as the example cytokine shown. **B.** Study day 0 Spike, CD4-RE, or Combined (Spike + CD4-RE) AIM^+ CD4 T cells by surface $OX40^+CD40L^+$. **C-E** Study day 0 Spike, CD4-RE, and combined total CD4 T cells by clinical trial assignment (bamlanivimab = "Treatment", saline = "Placebo") by (**C**) surface $OX40^+CD40L^+$, (**D**) $OX40^+41BB^+$, or (**E**) surface CD40L and intracellular IFNγ. **F-G.** Study day 0 Spike, CD4-RE, and combined circulating TFH cells by surface (**F**) $OX40^+41BB^+$ or (**G**) $OX40^+CD40L^+$. **H-J**. Example flow cytometry gating and frequencies for total CD4 T cells based on surface $CD40L^+$ and intracellular production of (**H**) GzmB, (**I**) TNF, (**J**) IL-2. **K-M.** % SARS2-specific of total CD4 T cells for MP versus DMSO stimulated by (**K**) $OX40^+41BB^+$ (**L**) $OX40^+CD40L^+$, and (**M**) $CD40L^+IFN\gamma^+$. **N-P.** Spike, non-Spike (CD4-RE) and total (Spike + CD4-RE = Combined) SARS2-specific CD4 T cells by (**N**) $OX40^+41BB^+$, (**O**) $OX40^+CD40L^+$, and (**P**) $CD40L^+IFN\gamma^+$ by stimulation index (SI, fold-change relative to DMSO control; see Methods for details). Bars, dotted lines, and flow cytometry gate labels as in **Fig. 2**. ns = not significant between Treatment and Placebo group by Mann-Whitney for comparison of the same stimulation condition. **** = $p \leq 0.0001$ by Mann-Whitney. **Related to Figure 2.**

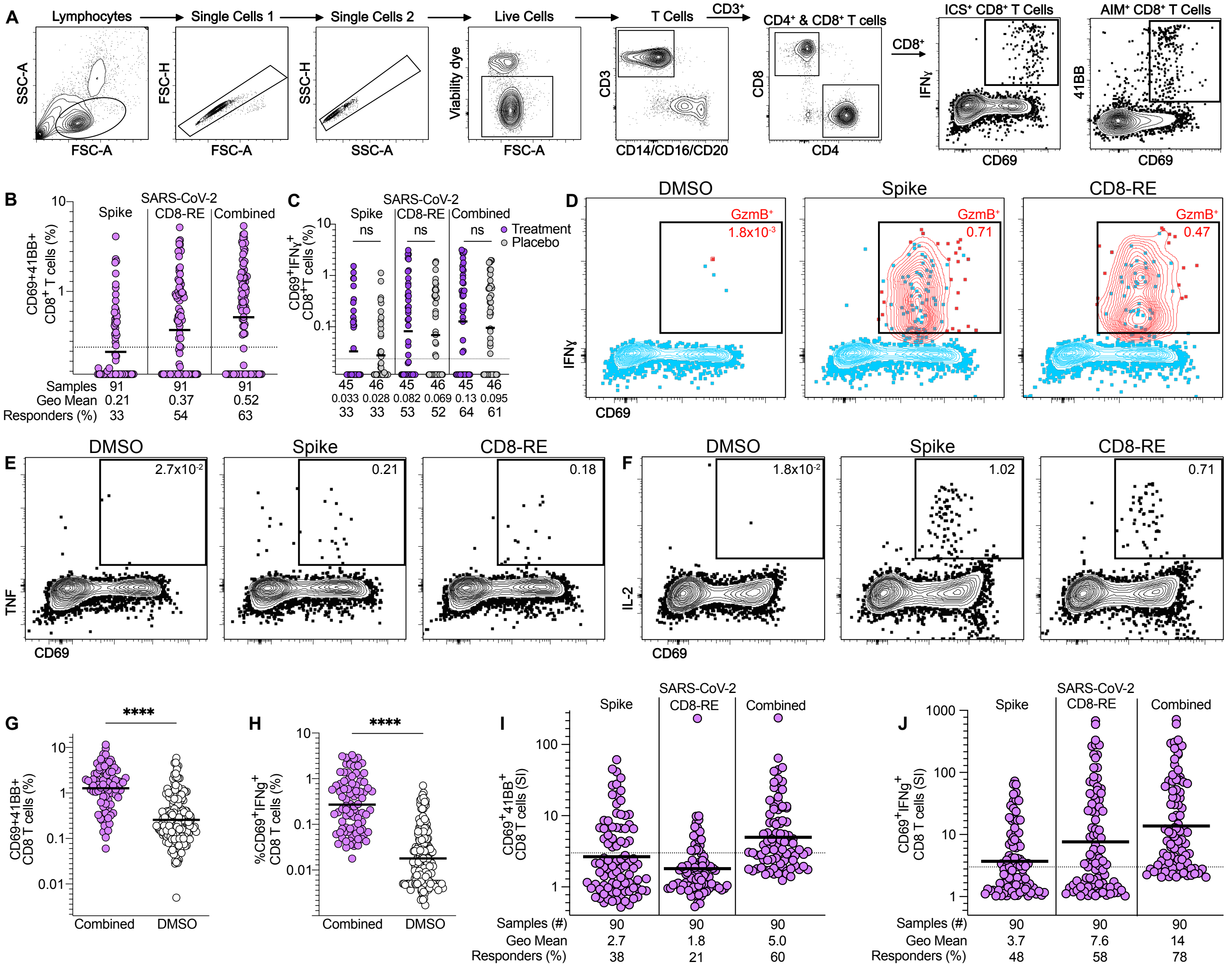

Figure S3. Antigen-specific CD8 T cell responses to primary SARS2 infection and acute COVID-19.

A. Gating strategy and representative flow cytometry gating for CD8 AIM and main ICS assay readouts. **B-C.** Study day 0 Spike, CD8-RE, or Combined CD8 responses by (**B**) AIM (CD69$^+$41BB$^+$) for all participants or (**C**) main ICS readout (CD69$^+$IFNγ$^+$) by clinical trial assignment (bamlanivimab = "Treatment", saline = "Placebo"). Bars and lines as in **Fig. 3**. ns = not significant between Treatment and Placebo group by Mann-Whitney for comparison of the same stimulation condition. **D-F.** Representative flow cytometry gating and frequencies for other intracellular cytokine production by SARS2-specific surface CD69$^+$ CD8 T cells for (**D**) GzmB (frequency of triple positive red cells out of total CD69$^+$IFNγ$^+$; other cells shown in light blue), (**E**) TNF, (**F**) IL-2. **G-H.** % SARS2-specific of total CD8 T cells for MP stimulated conditions versus DMSO control conditions by (**G**) CD69$^+$41BB$^+$ and (**H**) CD69$^+$IFNγ$^+$. **I-J.** Spike, non-Spike (CD8-RE) and total (Spike + CD8-RE = Combined) SARS2-specific CD8 T cells by (**I**) CD69$^+$41BB$^+$ and (**J**) CD69$^+$IFNγ$^+$ by stimulation index (SI, fold-change relative to DMSO control; see Methods). ns = not significant, **** = p ≤ 0.0001 by Mann-Whitney. **Related to Figure 3.**

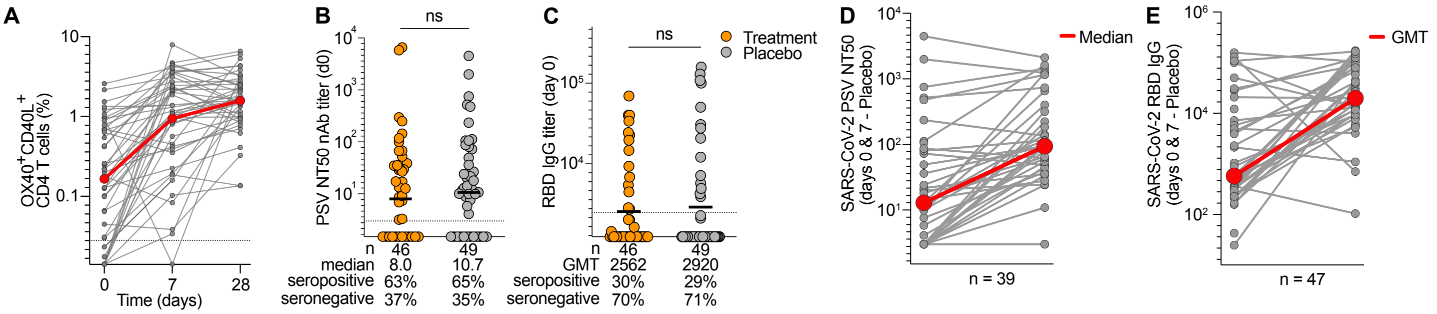

Figure S4. Humoral responses to primary SARS2 infection and acute COVID-19.

A. Longitudinal AIM$^+$ CD4 T cell responses by surface OX40$^+$CD40L$^+$ in the placebo group (n = 49) at study days 0, 7, and 28. Red line is median for each time point. **B-C.** Day 0 (**B**) nAb titers and (**C**) RBD IgG titers by clinical trial assignment (bamlanivimab = "Treatment", saline = "Placebo"). **D-E.** Day 0 and 7 (**D**) nAb titers and (**E**) RBD IgG titers for the placebo group (n = 49) participants. Bars and lines as in **Fig. 4** unless labeled differently. **Related to Figure 4.**

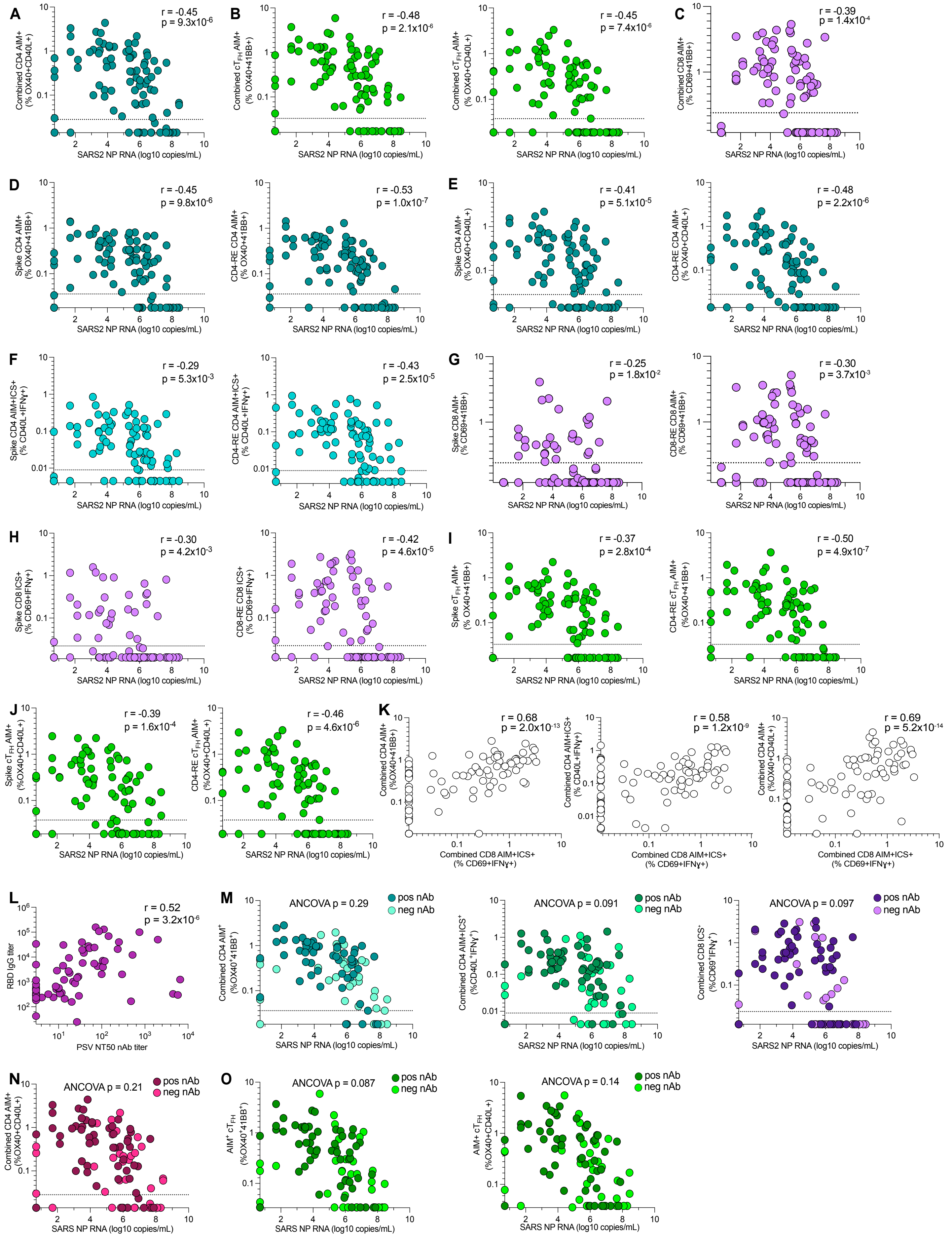

A
r = -0.45
p = 9.3x10^-6
Combined CD4 AIM+ (% OX40+CD40L+)
SARS2 NP RNA (log10 copies/mL)
B
r = -0.48
p = 2.1x10^-6
Combined cTFH AIM+ (% OX40+41BB+)
r = -0.45
p = 7.4x10^-6
Combined cTFH AIM+ (% OX40+CD40L+)
C
r = -0.39
p = 1.4x10^-4
Combined CD8 AIM+ (% CD69+41BB+)
D
r = -0.45
p = 9.8x10^-6
Spike CD4 AIM+ (% OX40+41BB+)
r = -0.53
p = 1.0x10^-7
CD4-RE CD4 AIM+ (% OX40+41BB+)
E
r = -0.41
p = 5.1x10^-5
Spike CD4 AIM+ (% OX40+CD40L+)
r = -0.48
p = 2.2x10^-6
CD4-RE CD4 AIM+ (% OX40+CD40L+)
F
r = -0.29
p = 5.3x10^-3
Spike CD4 AIM+ICS+ (% CD40L+IFNγ+)
r = -0.43
p = 2.5x10^-5
CD4-RE CD4 AIM+ICS+ (% CD40L+IFNγ+)
G
r = -0.25
p = 1.8x10^-2
Spike CD8 AIM+ (% CD69+41BB+)
r = -0.30
p = 3.7x10^-3
CD8-RE CD8 AIM+ (% CD69+41BB+)
H
r = -0.30
p = 4.2x10^-3
Spike CD8 ICS+ (% CD69+IFNγ+)
r = -0.42
p = 4.6x10^-5
CD8-RE CD8 ICS+ (% CD69+IFNγ+)
I
r = -0.37
p = 2.8x10^-4
Spike cTFH AIM+ (% OX40+41BB+)
r = -0.50
p = 4.9x10^-7
CD4-RE cTFH AIM+ (%OX40+41BB+)
J
r = -0.39
p = 1.6x10^-4
Spike cTFH AIM+ (%OX40+CD40L+)
r = -0.46
p = 4.6x10^-6
CD4-RE cTFH AIM+ (%OX40+CD40L+)
K
r = 0.68
p = 2.0x10^-13
Combined CD4 AIM+ (%OX40+41BB+)
Combined CD8 AIM+ICS+ (% CD69+IFNγ+)
r = 0.58
p = 1.2x10^-9
Combined CD4 AIM+ICS+ (% CD40L+IFNγ+)
r = 0.69
p = 5.2x10^-14
Combined CD4 AIM+ (%OX40+CD40L+)
L
r = 0.52
p = 3.2x10^-6
RBD IgG titer
PSV NT50 nAb titer
M
ANCOVA p = 0.29
pos nAb
neg nAb
Combined CD4 AIM+ (%OX40+41BB+)
SARS NP RNA (log10 copies/mL)
ANCOVA p = 0.091
Combined CD4 AIM+ICS+ (%CD40L+IFNγ+)
ANCOVA p = 0.097
Combined CD8 ICS+ (%CD69+IFNγ+)
N
ANCOVA p = 0.21
Combined CD4 AIM+ (%OX40+CD40L+)
O
ANCOVA p = 0.087
AIM+ cTFH (%OX40+41BB+)
ANCOVA p = 0.14
AIM+ cTFH (%OX40+CD40L+)

Figure S5. Unadjusted correlative relationships between virus-specific immune responses and upper airway SARS2 RNA levels. A-D. Correlation analyses similar to **Fig. 5A-C** but for study day 0 SARS2 NP RNA and SARS2-specific T cell responses by (**A**) CD4 T cell $OX40^+CD40L^+$ AIM; (**B**) circulating T_{FH} by $OX40^+41BB^+$ AIM or $OX40^+CD40L^+$ AIM; (**C**) CD8 T cell $CD69^+41BB^+$ AIM. **D-J.** Relationships for study day 0 SARS2 NP RNA and SARS2-specific T cell responses as in **Fig. 5A-C** and **Fig. S5A-C** split by responses to S or non-S MP stimulation for (**D**) CD4 T cell $OX40^+41BB^+$ AIM (**E**) CD4 T cell $OX40^+CD40L^+$ AIM, (**F**) CD4 T cell $CD40L^+IFN\gamma^+$, (G) CD8 T cell $CD69^+41BB^+$ AIM, (**H**) CD8 T cell $CD69^+IFN\gamma^+$, (**I**) circulating T_{FH} by $OX40^+41BB^+$ AIM, (**J**) circulating T_{FH} by $OX40^+CD40L^+$ AIM. **K.** Relationships between study day 0 SARS2-specific combined CD4 and CD8 responses by AIM and/or ICS. **L.** Relationship between day 0 nAb and RBD IgG titers. **M-O.** Impact of study day 0 nAb serostatus on relationships between study day 0 T cell responses and SARS2 NP RNA shown in **Fig. S5B** (**O** for circulating T_{FH}), **Figs. 5A-C** and **Fig. S5A** (**M** and **N** for total CD4 and CD8) by ANCOVA. All correlations by two-tailed nonparametric testing; r = Spearman's rank correlation coefficient. **Related to Figure 5.**

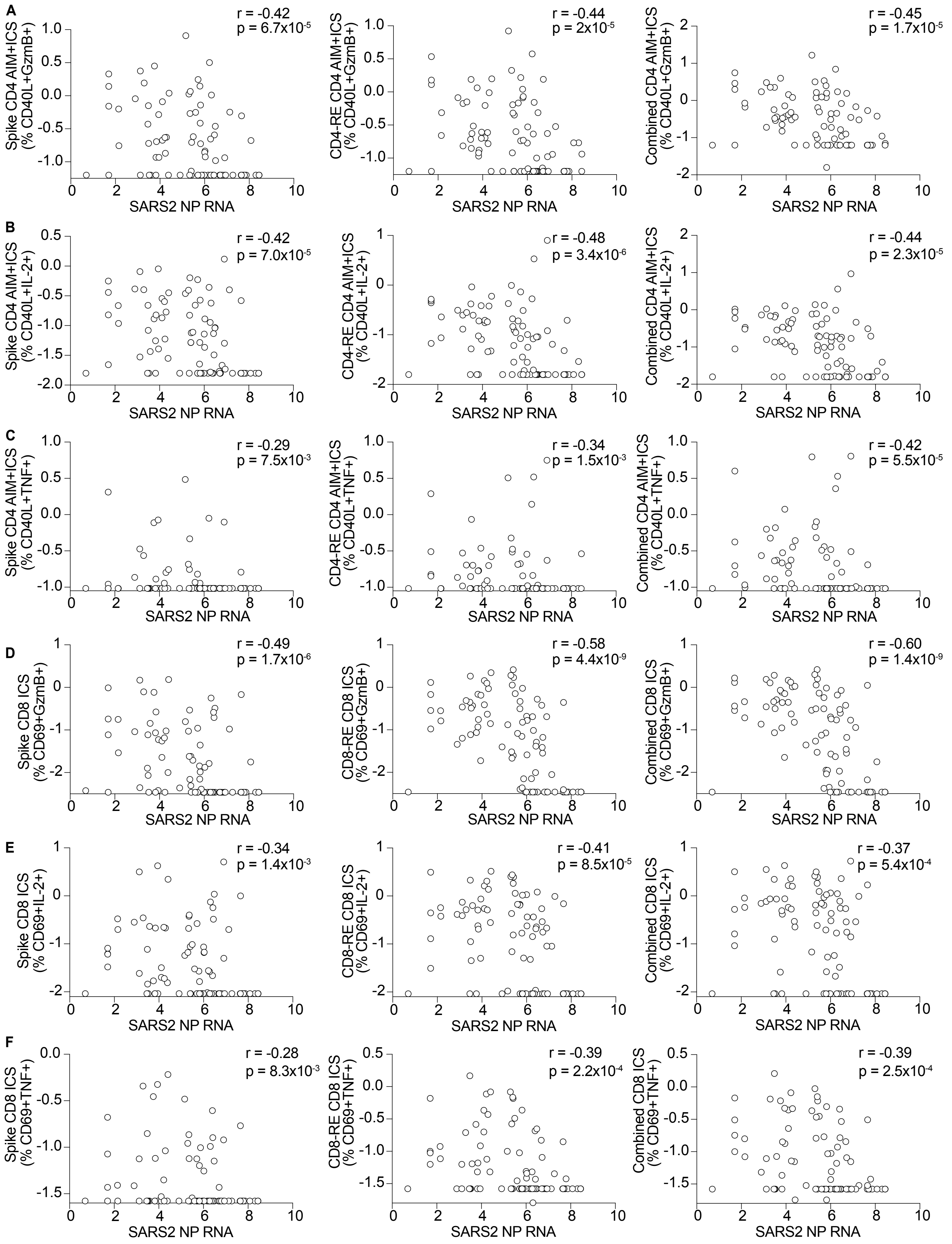
A
r = -0.42
p = 6.7x10^-5
Spike CD4 AIM+ICS (% CD40L+GzmB+)
SARS2 NP RNA
r = -0.44
p = 2x10^-5
CD4-RE CD4 AIM+ICS (% CD40L+GzmB+)
SARS2 NP RNA
r = -0.45
p = 1.7x10^-5
Combined CD4 AIM+ICS (% CD40L+GzmB+)
SARS2 NP RNA
B
r = -0.42
p = 7.0x10^-5
Spike CD4 AIM+ICS (% CD40L+IL-2+)
SARS2 NP RNA
r = -0.48
p = 3.4x10^-6
CD4-RE CD4 AIM+ICS (% CD40L+IL-2+)
SARS2 NP RNA
r = -0.44
p = 2.3x10^-5
Combined CD4 AIM+ICS (% CD40L+IL-2+)
SARS2 NP RNA
C
r = -0.29
p = 7.5x10^-3
Spike CD4 AIM+ICS (% CD40L+TNF+)
SARS2 NP RNA
r = -0.34
p = 1.5x10^-3
CD4-RE CD4 AIM+ICS (% CD40L+TNF+)
SARS2 NP RNA
r = -0.42
p = 5.5x10^-5
Combined CD4 AIM+ICS (% CD40L+TNF+)
SARS2 NP RNA
D
r = -0.49
p = 1.7x10^-6
Spike CD8 ICS (% CD69+GzmB+)
SARS2 NP RNA
r = -0.58
p = 4.4x10^-9
CD8-RE CD8 ICS (% CD69+GzmB+)
SARS2 NP RNA
r = -0.60
p = 1.4x10^-9
Combined CD8 ICS (% CD69+GzmB+)
SARS2 NP RNA
E
r = -0.34
p = 1.4x10^-3
Spike CD8 ICS (% CD69+IL-2+)
SARS2 NP RNA
r = -0.41
p = 8.5x10^-5
CD8-RE CD8 ICS (% CD69+IL-2+)
SARS2 NP RNA
r = -0.37
p = 5.4x10^-4
Combined CD8 ICS (% CD69+IL-2+)
SARS2 NP RNA
F
r = -0.28
p = 8.3x10^-3
Spike CD8 ICS (% CD69+TNF+)
SARS2 NP RNA
r = -0.39
p = 2.2x10^-4
CD8-RE CD8 ICS (% CD69+TNF+)
SARS2 NP RNA
r = -0.39
p = 2.5x10^-4
Combined CD8 ICS (% CD69+TNF+)
SARS2 NP RNA

Figure S6. Additional unadjusted correlative relationships between SARS2-specific T cell cytokine production and SARS2 NP RNA levels. A-C. Correlation analyses for study day 0 SARS2 NP RNA and SARS2 Spike (left), non-Spike (CD4-RE; middle), and combined (right) CD4 T cell cytokine production by CD4 T cell AIM+ICS: (**A**) CD40L^{+}GzmB^{+} (**B**) CD40L^{+}IL-2^{+} (**C**) CD40L^{+}TNF^{+}. **D-F.** Correlation analyses for study day 0 SARS2 NP RNA and SARS2 Spike (left), non-Spike (CD8-RE; middle), and combined (right) CD8 T cell cytokine production by CD8 T cell ICS: (**D**) CD69^{+}IFNγ^{+}GzmB^{+}, (**E**) CD69^{+}IL-2^{+}, (**F**) CD69^{+}TNF^{+}. All correlations by two-tailed nonparametric testing; r = Spearman's rank correlation coefficient. **Related to Figure 5.**

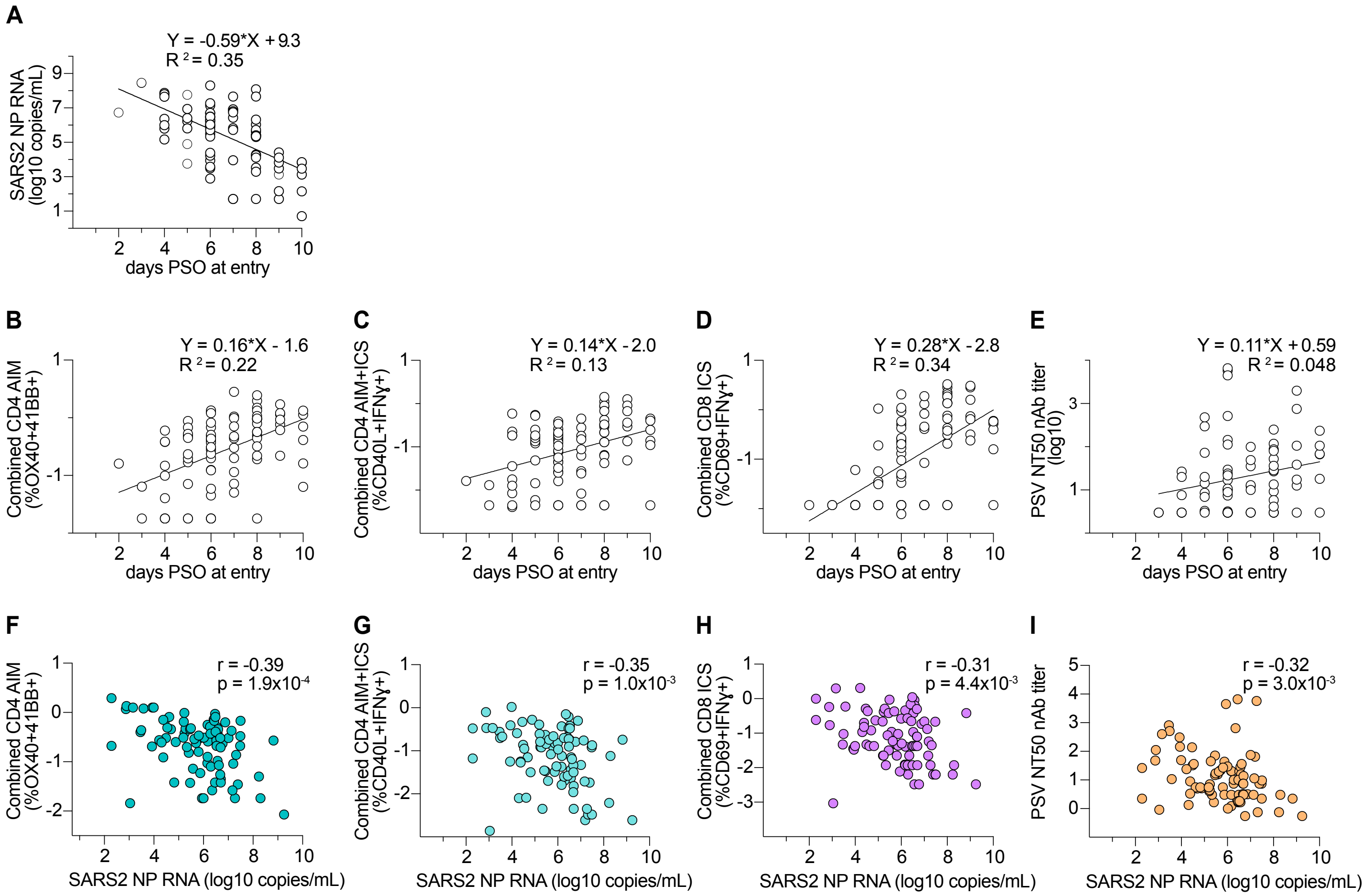

Figure S7. Adjusted correlative relationships between virus-specific immune responses and upper airway SARS2 RNA levels.
A-E. Log-linear regression models to account for variance in days from COVID-19 symptom onset to study entry (study day 0) for (A) SARS2 NP viral RNA, (B-C) SARS2-specific CD4 T cell responses, (D) SARS2-specific CD8 T cell responses, (E) and SARS2 nAb titers. Days post-symptom onset (PSO) at study day 0 values were plotted versus (A) SARS2 NP viral RNA or (B-E) immune responses at study day 0. Linear regression equations used for adjusted correlation analyses in **F-I** are shown. All immune response values in B-E were transformed to $\log_{10}$. **F-I.** Correlation analyses as in **Fig. 5A-D** for SARS2 NP RNA and SARS2-specific T cell responses by (**F**) CD4 AIM$^+$, (**G**) CD4 IFNγ, (**H**) CD8 IFNγ, and (**I**) nAb titers. In **F-I**, adaptive immune response values were adjusted to day 6 PSO using the related log-linear regression lines shown in **B-E** and plotted versus the corresponding adjusted day 6 PSO SARS2 NP RNA values generated from **A**. All correlations by two-tailed nonparametric testing; r = Spearman's rank correlation coefficient. **Related to Figure 5.**

SUPPLEMENTAL ACKNOWLEDGEMENTS

ACTIV-2/A5401 Study Team

Kara Chew, MD, MS, Co-Chair, David Geffen School of Medicine at University of California, Los Angeles, Los Angeles, CA, USA

David (Davey) Smith, MD, MAS, Co-Chair, University of California, San Diego, La Jolla, CA, USA

Eric Daar, MD, Vice Chair, Lundquist Institute at Harbor-UCLA Medical Center, Torrance, CA, USA

David Wohl, MD, Vice Chair, University of North Carolina at Chapel Hill School of Medicine, Chapel Hill NC, USA

Judith Currier, MD, MSc, Protocol Investigator and ACTG Chair, David Geffen School of Medicine at University of California, Los Angeles, Los Angeles, CA, USA

Joseph Eron, MD, Protocol Investigator and ACTG Vice Chair, University of North Carolina at Chapel Hill School of Medicine, Chapel Hill NC, USA

Arzhang Cyrus Javan, MD, MPH, DTM&H, NIH Division of AIDS (DAIDS) Clinical Representative, National Institutes of Health, Rockville, MD, USA

Michael Hughes, PhD, Lead Statistician, Harvard T.H. Chan School of Public Health, Boston, MA, USA

Carlee Moser, PhD, Statistician, Harvard T.H. Chan School of Public Health, Boston, MA, USA

Mark Giganti, PhD, Statistician, Harvard T.H. Chan School of Public Health, Boston, MA, USA

Justin Ritz, MS, Statistician, Harvard T.H. Chan School of Public Health, Boston, MA, USA

Lara Hosey, MA, Clinical Trials Specialist, AIDS Clinical Trials Group (ACTG) Network Coordinating Center, Social & Scientific Systems, a DLH Company, Silver Spring, MD, USA

Jhoanna Roa, MD, Clinical Trials Specialist, AIDS Clinical Trials Group (ACTG) Network Coordinating Center, Social & Scientific Systems, a DLH Company, Silver Spring, MD, USA

Nilam Patel, Clinical Trials Specialist, AIDS Clinical Trials Group (ACTG) Network Coordinating Center, Social & Scientific Systems, a DLH Company, Silver Spring, MD, USA

Kelly Colsh, PharmD, DAIDS Pharmacist, NIH/DAIDS Pharmaceutical Affairs Branch, Rockville, MD, USA

Irene Rwakazina, PharmD, DAIDS Pharmacist, NIH/DAIDS Pharmaceutical Affairs Branch, Rockville, MD, USA

Justine Beck, PharmD, DAIDS Pharmacist, NIH/DAIDS Pharmaceutical Affairs Branch, Rockville, MD, USA

Scott Sieg, PhD, Protocol Immunologist, Case Western Reserve University, Cleveland, OH, USA

Jonathan Li, MD, MMSc, Protocol Virologist, Brigham and Women's Hospital, Harvard Medical School, Boston, MA, USA

Courtney Fletcher, PharmD, Protocol Pharmacologist, University of Nebraska Medical Center, Omaha, NE, USA

William Fischer MD, Protocol Critical Care Specialist, University of North Carolina at Chapel Hill School of Medicine, Chapel Hill NC, USA

Teresa Evering, MD, MS, Protocol Investigator, Weill Cornell Medicine, New York, NY, USA

Rachel Bender Ignacio, MD, MPH, Protocol Investigator, University of Washington, Seattle, WA, USA

Sandra Cardoso, MD, PhD, Protocol Investigator, Fundação Oswaldo Cruz, Rio de Janeiro, Brazil

Katya Corado, MD, Lundquist Institute at Harbor-UCLA Medical Center, Torrance, CA, USA

Prasanna Jagannathan, MD, Protocol Investigator, Stanford University School of Medicine, Palo Alto, CA, USA

Nikolaus Jilg, MD, PhD, Protocol Investigator, Massachusetts General Hospital, Harvard Medical School, Boston, MA, USA

Alan Perelson, PhD, Protocol Investigator, Los Alamos National Laboratory, Los Alamos, NM, USA

Sandy Pillay, MB, CHB, Protocol Investigator, Enhancing Care Foundation, Durban, KwaZulu-Natal, South Africa

Cynthia Riviere, MD, Protocol Investigator, GHESKIO Center, Port-au-Prince, Haiti

Upinder Singh, MD, Protocol Investigator, Stanford University School of Medicine, Palo Alto, CA, USA

Babafemi Taiwo, MBBS, MD, Protocol Investigator, Northwestern University Feinberg School of Medicine, Chicago, IL, USA

Joan Gottesman, BSN, RN, CCRP, Field Representative, Vanderbilt University Medical Center, Nashville, TN, USA

Matthew Newell, BSN, RN, CCRN, Field Representative, University of North Carolina at Chapel Hill School of Medicine, Chapel Hill NC, USA

Susan Pedersen, BSN, RN, Field Representative, University of North Carolina at Chapel Hill School of Medicine, Chapel Hill NC, USA

Joan Dragavon, MLM, Laboratory Technologist, University of Washington, Seattle, WA, USA

Cheryl Jennings, BS, Laboratory Technologist, Northwestern University, Chicago, IL, USA

Brian Greenfelder, BA, Laboratory Technologist, Ohio State University, Columbus, OH, USA

William Murtaugh, MPH, Laboratory Specialist, ACTG Laboratory Center, University of California, Los Angeles, Los Angeles, CA, USA

Jan Kosmyna, MIS, RN, CCPR, ACTG Community Scientific Subcommittee Representative, Case Western University Clinical Research Site, North Royalton, OH, USA

Morgan Gapara, MPH, International Site Specialist, ACTG Network Coordinating Center, Social & Scientific Systems, a DLH Company, Durham, NC, USA

Akbar Shahkolahi, PhD, International Site Specialist, ACTG Network Coordinating Center, Social & Scientific Systems, a DLH Company, Silver Spring, MD, USA

Paul Klekotka, MD, PhD, Industry Representative, Lilly Research Laboratories, San Diego, CA, USA

SUPPLEMENTAL TABLES

Table S1. Antibodies used (from Ref. 21)

Marker-Fluorophore	Clone	Vendor	Catalog #	Assay
Fixable live/dead blue	N/A	Thermo Fisher	L34962	AIM, AIM+ICS
CD3-BUV395	UCHT1	BD	563546	AIM, AIM+ICS
CD4-cFluor B548	SK3	Cytek	SKU R7-20044	AIM, AIM+ICS
CD8a-BUV805	SK1	BD	612889	AIM, AIM+ICS
CD14/CD16/CD20-BV510	63D3/3G8/2H7	BioLegend	367123/302048/302340	AIM, AIM+ICS
CD45RA- BV570	HI100	BioLegend	304132	AIM, AIM+ICS
CCR7-BV711	G043H7	BioLegend	353228	AIM
CCR7-PE-Cy7	G043H7	BioLegend	353226	AIM+ICS
OX40-APC	Ber-Act35	BioLegend	350008	AIM
OX40-APC/Fire750	Ber-Act35	BioLegend	350031	AIM+ICS
CD137-BUV737	4B4-1	BD	568348	AIM
CD137-PE-Cy5	4B4-1	BioLegend	309808	AIM+ICS
CD40L-PE/Dazzle594	24-31	Biolegend	310840	AIM
CD40L-PerCP-eF710	24-31	Thermo	46-1548-42	AIM+ICS
CD25-APC/Fire750	BC96	BioLegend	302642	AIM
CD25-BV650	BC96	BioLegend	302634	AIM+ICS
CD69-FITC	FN50	BioLegend	310904	AIM
CD69-BV605	FN50	BioLegend	310938	AIM+ICS
CCR6-BUV496	11A9	BD	612948	AIM
ICOS-BUV563	DX29	BD	741421	AIM
CXCR5-BV421	J252D4	BioLegend	356920	AIM
CXCR3-BV605	G025H7	BioLegend	353728	AIM
CD38-BV650	HB-7	BioLegend	356620	AIM
PD-1-BV785	EH12.2H7	BioLegend	329930	AIM
CD95	DX2	BD	566542	AIM
PD-L1-PE	29E.2A3	BioLegend	329706	AIM
HLA-DR-APC-R700	Tu39	BD	746979	AIM
CCR4-PE-Cy7	L291H4	BioLegend	359410	AIM+ICS
IFNγ-AF488	4S.B3	BioLegend	502515	AIM+ICS
IL-2-BB700	MQ1-17H12	BD	566405	AIM+ICS
IL-4-BUV737	MP4-25D2	BD	612835	AIM+ICS
IL-10-PE/Dazzle594	JES3-19F1	BioLegend	506812	AIM+ICS
IL-17a-BV785	BL168	BioLegend	512338	AIM+ICS
IL-21-PE	4BG1	BioLegend	516704	AIM+ICS
GzmB-AF647	GB11	Biolegend	515406	AIM+ICS
TNFα-eF450	Mab11	eBioscience	48-7349-42	AIM+ICS
